# Drought conditions reorder plant communities through expansion of spatially sparse and temporally intermittent species

**DOI:** 10.1002/ecy.70478

**Published:** 2026-09-08

**Authors:** Carmen R. E. Watkins, Beatriz A. Aguirre, Y. Anny Chung, Lukas P. Bell‐Dereske, Lauren M. Hallett, David L. Hoover, Laureano A. Gherardi, Joan C. Dudney, Megan E. Wilcots, Jennifer A. Rudgers, Scott L. Collins, Melinda D. Smith, Forest Isbell, Tadashi Fukami, Hanan Farah, Cristina Portales‐Reyes

**Affiliations:** ^1^ Department of Biology University of Oregon Eugene Oregon USA; ^2^ Department of Ecology and Evolutionary Biology Cornell University Ithaca New York USA; ^3^ Department of Plant Biology and Department of Plant Pathology University of Georgia Athens Georgia USA; ^4^ Institute of Microbiology of the Czech Academy of Sciences Prague Czech Republic; ^5^ Environmental Studies Program University of Oregon Eugene Oregon USA; ^6^ Rangeland Resources and Systems Research Unit USDA Agricultural Research Service Fort Collins Colorado USA; ^7^ Department of Environmental Science, Policy, and Management University of California, Berkeley Berkeley California USA; ^8^ Environmental Studies Program University of California Santa Barbara Santa Barbara California USA; ^9^ Bren School of Environmental Science and Management University of California Santa Barbara Santa Barbara California USA; ^10^ The Nature Conservancy Minneapolis Minnesota USA; ^11^ Department of Biology University of New Mexico Albuquerque New Mexico USA; ^12^ Department of Biology and Graduate Degree Program in Ecology Colorado State University Fort Collins Colorado USA; ^13^ Department of Ecology, Evolution and Behavior University of Minnesota St. Paul Minnesota USA; ^14^ Department of Biology Stanford University Stanford California USA; ^15^ Department of Earth System Science Stanford University Stanford California USA; ^16^ Department of Biology Saint Louis University St. Louis Missouri USA; ^17^ Present address: Department of Botany Trinity College Dublin Dublin Ireland

**Keywords:** common species, drought recovery, legacy effects, rare species, spatial rarity, temporal rarity

## Abstract

The frequency and severity of droughts are increasing in grasslands globally. Forecasting changes in community composition is challenging due to the direct and indirect effects of drought as well as varied species ecologies. Rare species are predicted to be more susceptible to perturbations, but extreme drought may also disproportionately reduce common species that are well adapted to modal environmental conditions. Resolving these contrasting effects is key to predicting species reordering in response to extreme drought. We explored how species drought responses varied by their spatial and temporal rarity, defined by their local abundances relative to co‐occurring species, at six sites across a gradient of mean annual precipitation (MAP) in the Central USA. We found that in general, both spatially sparse and temporally intermittent species increased during and after drought, while common and persistent species declined. Responses were similar at four of the six sites across a gradient of MAP, but did not align with the precipitation gradient as expected. Instead, patterns depended on the characteristics of common species, where rarity was not related to drought responses at sites with many common, early‐season, C_3_ annual grasses that likely avoided the effects of the summer drought. Finally, the increase in rare species persisted for 4 years after the experimental drought was lifted, suggesting that this extreme drought resulted in lasting shifts to community composition. These findings highlight that rare species can be favored under non‐modal environmental conditions like extreme drought and underscore the importance of assessing species recovery to periodic environmental disturbances.

## INTRODUCTION

The frequency and severity of droughts are increasing in grasslands globally with climate change (Cook et al., 2015; Dai, 2013), yet predictions of how drought will alter grassland plant communities are complicated by the varied ways that drought affects species performance. For example, drought often has a negative direct effect on physiology, but can also have a beneficial indirect effect by reducing interspecific competition (Cavin et al., 2013; Levine et al., 2010). These phenomena can occur both during the drought and after the drought subsides through legacy effects, in which slow physiological recovery, delayed propagule availability, or altered species interactions can lead to persistent changes in abundance after drought ends (Dudney et al., 2017; Sala et al., 2012; Vilonen et al., 2022). Species abundance distributions may yield insights into which species will have net negative or positive drought responses (Avolio et al., 2015). Rare species, for example, are predicted to be more sensitive to extreme events than common species due to stochasticity and small population sizes, and may be slow to recover post‐perturbation (Arnoldi et al., 2018; Duwyn & MacDougall, 2015; Pimm et al., 1988; Tilman & Haddi, 1992). On the other hand, common species are likely best adapted to modal environmental conditions (Gherardi & Sala, 2015) and their high biomass and frequency in the community may become a disadvantage when environmental conditions are not optimal and resources are limited under drought (Diez et al., 2021). Reconciling these contrasting perspectives on how and when rare or common species respond to drought may provide a general framework for anticipating drought effects on grassland plant community structure.

Within a local community, species abundance versus rarity can vary along two axes: space and time (Rabinowitz, 1981; Wilfahrt et al., 2021). Species abundance distributions are most frequently calculated across space, where spatially rare species are considered “sparse” and spatially abundant ones “common” (Hanski, 1982). However, species abundance versus rarity can also be conceptualized over time, such that temporally rare species can be considered “intermittent” and temporally abundant species “persistent” (Coyle et al., 2013; Wilfahrt et al., 2021). Spatial and temporal abundances are often but not always positively correlated (Magurran & Henderson, 2003) and may reflect different life histories, functional traits, or ecological strategies.

Exploring how spatial and temporal rarity relate to each other may provide novel insight into how different suites of species respond to drought. Spatially common species are often temporally persistent and well adapted to modal environmental conditions, as they have a strong linkage between the environment, their performance, and their traits (Castillioni & Isbell, 2023; Ge et al., 2019; Gherardi & Sala, 2015; Umaña et al., 2017). During drought, they may decline due to reduced environmental suitability or strong negative frequency dependence (Diez et al., 2021; Gherardi & Sala, 2015), although species with drought escape or tolerance mechanisms, such as a dormant bud bank, may recover quickly afterwards (Hoover et al., 2014). As a whole, small, sparse populations can be more vulnerable to demographic stochasticity, environmental fluctuations, and extreme events (Duwyn & MacDougall, 2015; Pimm et al., 1988). However, persistent, sparse species may be resource specialists in a narrow, stable niche or limited by strong interspecific competition (Hallett et al., 2018; McKane et al., 2002; Yenni et al., 2017), potentially allowing them to respond to competitive release during drought (Prugh et al., 2018). Temporally intermittent species have a weaker link between their traits and the environment than common, persistent species or may have unique temporal niches (Ge et al., 2019; Gherardi & Sala, 2015; Umaña et al., 2017), both of which may cause them to be spatially sparse as well. Finally, temporally intermittent species that can “store” resources during unfavorable periods (such as in the seed bank) and capitalize on resource pulses through time or space may become spatially common after drought (Angert et al., 2009).

The interaction between species rarity and the abiotic environment or species traits at a site may further influence species drought responses. For example, species in favorable environments with high water availability are expected to be most limited by competitive interactions, while species in low resource environments should be limited more by abiotic conditions (Louthan et al., 2015). As such, in mesic environments, drought may release rare species from competition by common species and in arid conditions, species may be sensitive to extreme drought regardless of their abundance (Huxman et al., 2004; Knapp et al., 2015; Mount et al., 2024; Smith et al., 2024). Conversely, species in arid systems may be well adapted to drought. Finally, the abiotic environment at a site also shapes community characteristics like the degree of dominance (Silva et al., 2010) and the species pool (Keddy, 1992). Higher dominance in a community should lead to greater competitive release if dominant species decline during drought (McCain et al., 2010), while filtering of the species pool may shape responses as certain traits are more prevalent in some environments, such as intermittent seed‐banking annuals in the desert or C_3_ species in colder environments (Ehleringer et al., 1997). Exploring drought responses and species characteristics across environmental gradients can tease apart whether broad scale environmental drivers or community characteristics drive drought responses.

To test immediate and legacy drought responses in relation to species abundance, we analyzed species cover data from the Extreme Drought in Grasslands Experiment (EDGE) in the central United States. EDGE experimentally imposed a severe drought for 4–7 years at six sites across a gradient of water availability as represented by mean annual precipitation (MAP) (Griffin‐Nolan et al., 2019). Communities were monitored for 4 years post‐drought, allowing us to examine species recovery, which is an understudied component of grassland responses to extreme disturbances (Isbell et al., 2015; Vilonen et al., 2022). We characterized the responses of 499 species populations and calculated their local rarity relative to co‐occurring species, to address the following questions: (1) Are spatially or temporally rare species more likely to capitalize on drought and post‐drought conditions than common species? And (2) Do environmental characteristics like water availability and temperature, or endogenous characteristics like species composition, alter rarity‐drought response patterns? We predicted that rare species would increase more during drought than common species, and that the type of rarity (spatial or temporal) would determine the strength of the response. Specifically, we expected that spatially rare species would increase during drought, possibly due to competitive release, while temporally rare species would increase most after drought when windows of reduced competition aligned with increased precipitation. We further expected larger increases in rare species abundances at mesic and intermediate precipitation sites due to competitive release from common species, compared to arid sites where species may be at the edge of their abiotic tolerances. Overall, linking species abundance and probable drought response trajectories will help predict species reordering within communities in response to severe drought conditions.

## METHODS

### Study system

**FIGURE 1:**
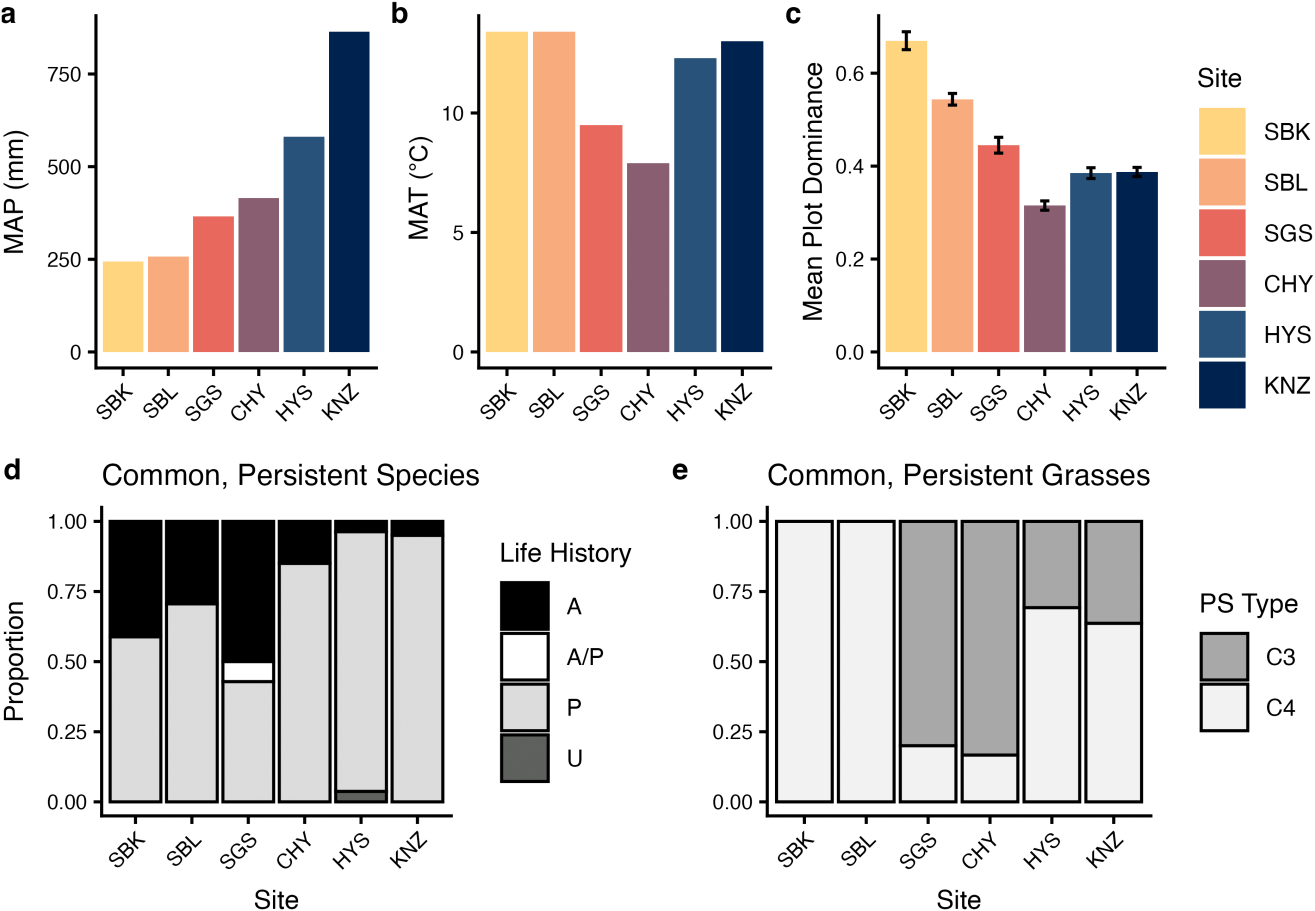
The mean annual precipitation (MAP) in millimeters (a), mean annual temperature (MAT) in degrees Celsius (b), and mean Berger–Parker dominance (c) at each site. The proportion of common, persistent species in each life history category by site (d) and the proportion of common, persistent grasses with C_3_ or C_4_ photosynthesis (PS) pathways (e). A, annual; P, perennial; U, unknown. Sites are as follows: CHY, High Plains Grasslands Research Center; HYS, Hays Agricultural Research Center; KNZ, Konza Prairie; SBK, Sevilleta Black Grama; SBL, Sevilleta Blue Grama; SGS, Short Grass Steppe.

We used species‐level, absolute percent cover data from six sites of the EDGE across the US: Konza Prairie (KNZ), Hays Agricultural Research Center (HYS), High Plains Grasslands Research Center (CHY), Short Grass Steppe (SGS), Sevilleta Blue Grama (SBL), and Sevilleta Black Grama (SBK). Sites span a west–east precipitation gradient and a north–south mean annual temperature (MAT) gradient (Figure 1a,b) and vary in soil water holding capacity (Appendix S1: Table S1) (Griffin‐Nolan et al., 2018; Wilcox et al., 2023). The biotic communities varied in the average level of dominance, the proportion of annual versus perennial species, and the proportion of common C_3_ versus C_4_ grasses (Figure 1c–e; Appendix S1: Figure S1).

Each site had 20 plots (6 × 6 m at KNZ, HYS, CHY, SGS, and 3 × 4 m at SBL, SBK) arranged into 10 blocks of two plots each. Within each block, one plot received ambient rainfall (*n* = 10), and the other was droughted (*n* = 10) during the growing season (April–mid‐September) using rainfall exclusion shelters that blocked 66% of ambient rainfall. Drought treatments lasted from 2014 to 2017 at KNZ, HYS, CHY, and SGS, and 2013 to 2019 at SBK and SBL, after which shelters were removed and post‐drought monitoring continued for 4 years, until 2021 and 2023, respectively. We assessed species composition during the spring and fall of each year using aerial percent cover within four 1 m^2^ subplots per plot. At SBK and SBL, we sampled four 1 m^2^ subplots from 2012 to 2018 and only two 1 m^2^ subplots from 2019 onward.

### Data processing

All analyses were conducted in R version 4.5.2 (R Core Team, 2025). We excluded 461 subplot level observations with unknown species identification from a total of 75,499 observations. When species identification was not consistent within a site, we consulted site experts to determine whether to lump, split, or remove taxa. Across our six sites, we retained 499 species populations (unique taxa × site combinations) after data processing. We selected the maximum cover of each species within each subplot in each year (spring or fall). When a species was found at a site, but there was no record of it in a particular subplot (rather than being recorded as 0), we filled in a 0 to ensure our spatial and temporal abundance measures captured all instances where a species could have been present.

### Calculating spatial and temporal rarity

We quantified local spatial rarity ($S_{R}$) for a given species *i* as 1 minus the proportional rank of species *i* in the community (Wilfahrt et al., 2021), using the percent_rank() function in the dplyr package (Wickham et al., 2023).

(1) $$S_{R,i}=1-\frac{\sum_{j}^{n}\left(\bar{C_{C,j}}<\bar{C_{C,i}}\right)}{n-1},$$

where $\bar{C_{C,j}}$ and $\bar{C_{C,i}}$ are the mean cover values of species *j* and *i* respectively, across all years in all control subplots at a site, and *n* is the number of species at a site. Summing $\bar{C_{C,j}}<\bar{C_{C,i}}$ for all species $j=\left[1,\ldots ,n\right]$ at a site returns the number of species with lower cover than species *i*, as the less than operator (<) evaluates as a Boolean function where TRUE = 1 and FALSE = 0. Spatial rarity ranges between 0 and 1, where values approaching 1 indicate rare species and values near 0 indicate species with high cover that appear in many plots. We measured local temporal rarity ($T_{R}$) for a given species *i* as

(2) $$T_{R,i}=1-\frac{y_{p,i}}{y_{t}},$$

where $y_{p,i}$ is the number of years that species *i* was present in *any* control plot at a site and $y_{t}$ is the total number of years in the time series (Wilfahrt et al., 2021). Temporal rarity also ranges from 0 to 1 where values close to 1 indicate temporally rare species that are present very few years and 0 indicates species that are present every year. We tested the correlation between spatial and temporal rarity with Pearson's correlation coefficient and found they were correlated. However, as the correlation varied between sites (Appendix S1: Figure S2), we retained both metrics in our analyses.

We used rarity as a continuous variable in most analyses, but categorized species into abundance typologies to explore the combined effects of spatial and temporal rarity on species' drought responses. We considered a species *spatially sparse* if $S_{R}$ ≥ 0.25 and *spatially common* if $S_{R}$ < 0.25. Similarly, species were defined as *temporally intermittent* if $T_{R}$ ≥ 0.5 and *temporally persistent* if $T_{R}$ < 0.5. There is no standard cutoff to delineate rare versus common species (Coyle et al., 2013), so we selected the spatial rarity cutoff of 0.25 based on right‐skewed rank abundance curves at each site and the temporal rarity cutoff as 0.5, as temporal rarity had a bimodal distribution centered near 0.5 at most sites (Appendix S1: Figure S3). Species therefore fell into four categories: *common* and *persistent* species that were not rare on either axis, species that were rare either spatially or temporally but not both (*sparse* but *persistent* species, or *common* but *intermittent* species), and finally *sparse* and *intermittent* species that were rare both spatially and temporally. To test how the bounds of each rarity category altered our results, we calculated the same categories, with cutoffs adjusted by ±0.10.

### Calculating drought and post‐drought responses

To assess each species response to drought, we calculated a response ratio (Armas et al., 2004):

(3) $${\mathrm{RR}}_{i}=\frac{C_{D,i}-C_{C,i}}{C_{D,i}+C_{C,i}},$$

where $C_{D}$ and $C_{C}$ are the mean cover values of a species *i* in the drought or control treatment, respectively, across all subplots and across all years during or after drought. This metric ranges from −1 to 1 and is positive when cover in drought plots is greater than controls and negative when cover is lower in drought plots than controls. We calculated a response ratio for each species at each site during the drought period and separately over the 4 years after drought. For sites SBL and SBK, which applied a 7‐year drought rather than a 4‐year drought, we used data from only the first 4 years of the drought to standardize among sites. Finally, to examine the experimental drought in the context of the existing rainfall conditions, we obtained the standardized precipitation–evapotranspiration index (SPEI) from SPEIbase version 2.11 (Beguería et al., 2024) for each year in each site, using 6‐month SPEI‐06 values from the end of the growing season (September at KNZ, HYS, CHY, and SGS or October at SBL and SBK).

### Statistical analyses

To test for the overall effect of rarity on a species response during and after drought, we used a linear mixed‐effects model with a fixed effect, rarity (continuous), and a random effect, site, as predictors of the response ratio, using the lme4 package (Bates et al., 2015).

(4) $$\text{Response Ratio}\sim \text{Rarity}+\left(1|\text{Site}\right).$$

We modeled spatial and temporal rarity in separate models for both the drought and post‐drought periods. We then tested the significance of effects using Type II ANOVA with the Anova() function in the Car package (Fox & Weisberg, 2019) and calculated the marginal ($R_{m}^{2}$) and conditional ($R_{c}^{2}$) *R* ^2^ values, where $R_{m}^{2}$ is the variance explained by the fixed effects in the model and $R_{c}^{2}$ is the variance explained by the full model (Nakagawa & Schielzeth, 2013).

We assessed whether the above relationship between rarity and drought response was robust to four data processing assumptions. First, we tested the impact of (1) removing unknown observations from our data, and (2) retaining transient species in our data (defined as not present in 1‐year pretreatment data and gained or lost), by separately relaxing each assumption, re‐calculating response ratios, and using Equation 4 to test whether rarity predicted drought responses. We then assessed if (3) calculating a response ratio across the post‐drought period was representative of recovery or if an early lack of recovery masked later trends, by splitting the post‐drought period into halves, calculating response ratio values for each half, and using a linear mixed‐effects model with additive, fixed effects of rarity and time period (first or final half) and a random effect of site to predict response ratios. Finally, we checked if (4) a 4‐year response ratio was representative of drought impacts at sites that were droughted for 7 years (SBL, SBK) by calculating response ratios over both 4 and 7 years and using a linear model with additive effects of rarity and drought length (4 or 7 years) to predict response ratios separately at SBL and SBK.

We further tested if life history (annual/perennial) influenced the relationship between rarity and response ratio using a linear mixed‐effects model with the fixed effects of rarity (continuous) and life history (categorical) as both main and interactive effects and a random effect of site as predictors of the response ratio.

(5) $$\text{Response Ratio}\sim \text{Rarity}\times \text{Life History}+\left(1|\text{Site}\right).$$

We tested the significance of the effects using Type III ANOVA to account for the interaction in the model, calculated $R_{m}^{2}$ and $R_{c}^{2}$ as above, and calculated the marginal slopes using the emtrends() function in the emmeans package (Lenth & Piaskowski, 2026). Finally, to explore if the effect of rarity varied at each site, we ran separate linear models for each site, rarity type, and drought versus post‐drought period combination with rarity as the predictor of the response ratio.

### Site‐level predictors

To explore mechanisms underlying site differences, we investigated additional site‐level predictors. We obtained long‐term MAP and MAT data for each site from Griffin‐Nolan et al. (2018), aridity data from Zomer et al. (2022), and soil field capacity data from Wilcox et al. (2023). To measure site‐level dominance by a single species, we calculated the Berger–Parker dominance of every control plot in every year at each site and took the average of these values (Berger & Parker, 1970). We calculated the z‐scores of predictors and used these values in linear models testing separately whether each predictor was related to the slope of the rarity‐response ratio relationship at each site. We retain MAP and MAT in our main analyses rather than using aridity, as aridity is closely correlated with MAP at our sites (Appendix S1: Figure S4). To characterize site differences in species growth forms, we compared the proportion of annual versus perennial species within each abundance typology (i.e., spatial and temporal rarity category), as well as the proportion of C_3_ versus C_4_ grass species.

## RESULTS

### Rarity predicted how species responded during and after drought

**FIGURE 2:**
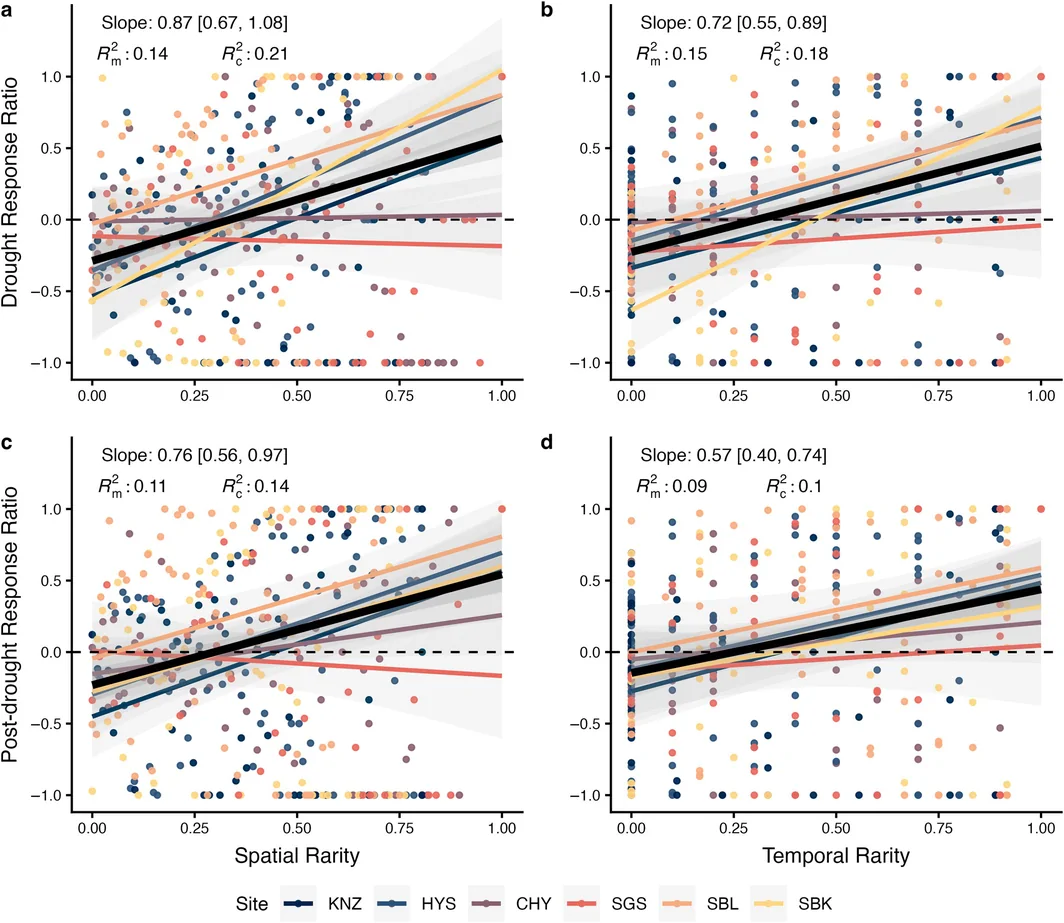
The effect of spatial (a, c) and temporal (b, d) rarity on a species' response ratio values during drought (a, b) and post‐drought (c, d). Each point shows one population of a species (i.e., species × site combination). Black lines indicate the overall response across sites, while colored lines indicate individual site responses. Positive values of the response ratio indicate cover of an individual species is greater in drought plots than controls and negative values occur when cover is lower in drought plots than controls. Spatial or temporal rarity values near 1 indicate rare species and values near 0 indicate common species. Slope and *R* ^2^ values at the top of panels are from linear mixed‐effects models testing the effect of rarity on drought response across sites (Equation 4). Slope values are shown with the 95% CI in brackets. Marginal $R_{m}^{2}$ = variation explained by fixed effects alone, conditional $R_{c}^{2}$ = variation explained by both fixed and random effects. Mixed‐effects model results are in Table 1 and individual site models are in Appendix S1: Table S8. Sites are as follows: CHY, High Plains Grasslands Research Center; HYS, Hays Agricultural Research Center; KNZ, Konza Prairie; SBK, Sevilleta Black Grama; SBL, Sevilleta Blue Grama; SGS, Short Grass Steppe.

Across all sites, rare species increased under drought while common species declined, regardless of whether rarity was temporal or spatial (Figure 2a,b). Common species declined by approximately 20%–30% during drought relative to controls, although the effect varied by site (average intercept among sites: $S_{R}=-0.31$, 95% CI [−0.49, −0.12], $T_{R}=-0.23$, 95% CI [−0.37, −0.09]). Overall, there was a positive relationship between species rarity and increasing abundance under drought ($S_{R}$: *F*_1,393_ = 72, *p* = 4.43 × 10^−16^; $T_{R}$: *F*_1,397_ = 70, *p* = 9.07 × 10^−16^; Table 1). Although this was a general trend, individual species varied considerably in their responses, and different life histories had broadly different patterns. Specifically, annual species often increased in cover during drought more than perennial species ($S_{R}$: *F*_1,388_ = 15, *p* = 0.0001; $T_{R}$: *F*_1,388_ = 4.5, *p* = 0.0345) and there was a significant interaction between spatial rarity and life history, such that common perennial species declined more steeply under drought than annuals ($S_{R}$: *F*_1,387_ = 11, *p* = 0.0009; Appendix S1: Figure S5, Tables S2 and S3).

**TABLE 1:** Fixed‐effect coefficients from linear mixed‐effects models testing the fixed effect of rarity on a species response ratio during and after drought, with a random effect of site to account for differences in sites (Equation 4).

Experimental period	Rarity type	Coefficient	Estimate	SE	df	*t* value	Pr(>|*t*|)
Drought	Spatial	Intercept	−0.31	0.09	8.97	−3.4	0.008
		Rarity	0.87	0.1	392.64	8.49	4.32 × 10^−16^
	Temporal	Intercept	−0.23	0.07	9.37	−3.26	0.009
		Rarity	0.72	0.09	396.78	8.42	7.14 × 10^−16^
Post‐Drought	Spatial	Intercept	−0.24	0.07	14.38	−3.17	0.007
		Rarity	0.76	0.1	419.22	7.33	1.22 × 10^−12^
	Temporal	Intercept	−0.14	0.06	15.34	−2.44	0.027
		Rarity	0.57	0.09	373.19	6.51	2.48 × 10^−10^

Abbreviations: df, degrees of freedom; SE, standard error.

Post‐drought, both spatially and temporally rare species tended to maintain their increased cover, whereas common species stayed at lower abundance in drought plots relative to controls ($S_{R}$: *F*_1,419_ = 54, *p* = 1.26 × 10^−12^; $T_{R}$: *F*_1,374_ = 41, *p* = 5.34 × 10^−10^; Figure 2c,d; Table 1). Common species still had lower cover compared to control levels, but intercept values were closer to zero than during drought, indicating some recovery (Table 1). Ambient precipitation fluctuations were similar during and after the experimentally imposed drought years, but SPEI values declined in the years following the experimental drought, potentially limiting recovery (Appendix S1: Figure S6).

### Rarity responses were robust to data processing assumptions

Generally, our results were robust to assumptions made during data processing. The main effect of rarity explained 14%–15% of the variation in our data, and it remained a significant predictor of drought responses across sites when *unknown species observations were included* in the dataset ($S_{R}$: *F*_1,411_ = 80, *p* < 2.20 × 10^−16^; $T_{R}$: *F*_1,414_ = 62, *p* = 3.26 × 10^−14^, Appendix S1: Figure S7, Table S4) and *when transient species*—defined as not present in 1‐year pretreatment data and gained or lost—*were removed* from the data set ($S_{R}$: *F*_1,333_ = 24, *p* = 1.26 × 10^−6^; $T_{R}$: *F*_1,331_ = 34, *p* = 1.05 × 10^−8^, Appendix S1: Figure S8, Table S5). Rarity explained only 7% ($S_{R}$) or 9% ($T_{R}$) of the variation in our data when transient species were removed, indicating that more transient species helped drive the pattern between rarity and drought response. The lack of recovery post‐drought was representative of the whole post‐drought period, as the relationship between rarity and drought response did not differ between the first or final 2 years of the post‐drought period ($S_{R}$: *F*_1,704_ = 2, *p* = 0.142; $T_{R}$: *F*_1,705_ = 2, *p* = 0.164; Appendix S1: Figure S9, Table S6). Finally, response ratio values were not significantly altered by the additional 3 years of experimental drought at sites SBL ($S_{R}$: *F*_1,138_ = 0.03, *p* = 0.870; $T_{R}$: *F*_1,138_ = 0.03, *p* = 0.865) and SBK ($S_{R}$: *F*_1,114_ = 0.01, *p* = 0.933; $T_{R}$: *F*_1,114_ = 0.0002, *p* = 0.989; Appendix S1: Figure S10, Table S7).

### Lag effects depended on abundance typology

**FIGURE 3:**
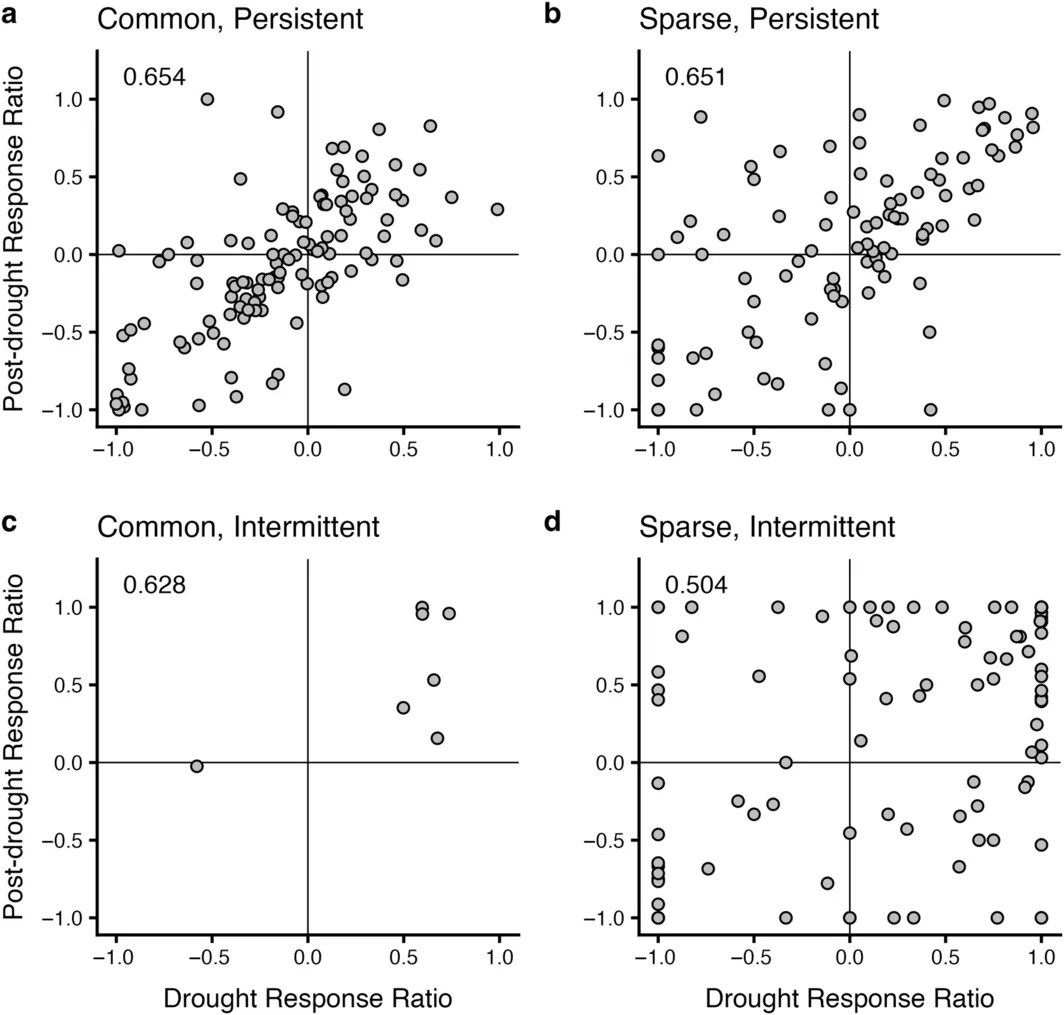
The drought response ratio plotted against the post‐drought response ratio for each species, separated into panels by the abundance typology of the species: Common, persistent species (a), sparse, persistent species (b), common, intermittent species (c), and sparse, intermittent species (d). Abundance typologies were defined by the combination of a species' spatial and temporal rarity, where spatial rarity is categorized into common ($S_{R}<0.25$) versus sparse ($S_{R}\geq 0.25$) species and temporal rarity into persistent ($T_{R}<0.5$) versus intermittent ($T_{R}\geq 0.5$) species. Each point shows one population of a species (i.e., species × site combination). Pearson's correlation coefficients for the relationship between drought and post‐drought response ratios are shown in the top left corner of each panel.

Spatially and temporally rare species often responded similarly during and after drought, as species spatial and temporal rarity values were positively correlated (Appendix S1: Figure S2). Spatially common and temporally persistent species were the most likely to respond the same way both during and after drought, whether positive or negative (*r* = 0.654, Figure 3a). These common persistent species were most often perennial, except at site SGS, and had a large proportion of grasses relative to other categories (Appendix S1: Figure S1). Spatially sparse and temporally persistent species also often shared immediate and legacy responses to drought (*r* = 0.651), but some that responded negatively during drought returned to or overshot control abundances (Figure 3b). Sparse, persistent species in contrast to common species were largely forbs, except at sites SBL and SBK, which had a high proportion of shrubs (including sub‐shrubs) in this category (Appendix S1: Figure S1). Spatially common, temporally intermittent species occurred only at sites SBL and SBK, and all increased both during and after drought, except *Euphorbia serpyllifolia* at site SBK (Figure 3c). Most of these species were annuals and forbs (Appendix S1: Figure S1). Finally, some spatially sparse and temporally intermittent species increased while others declined, but this did not predict post‐drought responses (*r* = 0.504, Figure 3d). Most sparse, intermittent species were forbs and perennials, although annual species made up a relatively large proportion of these species, 18%–47% (Appendix S1: Figure S1).

Defining common, persistent species more stringently increased the correlation between responses during and after drought from 0.654 to 0.761 (Appendix S1: Figure S11a), indicating that the most abundant and persistent species had the strongest inertia in their responses. Conversely, correlations between sparse species responses during and after drought changed only slightly under this definition (Appendix S1: Figure S11b–d). Broadening the definition of common, persistent species and thereby considering rarity more narrowly produced a smaller increase in correlation of responses in sparse, persistent species (from 0.651 to 0.661) and in sparse, intermittent species (from 0.504 to 0.534) (Appendix S1: Figure S11e–h).

### Responses of spatially and temporally rare species to drought differ by site

**FIGURE 4:**
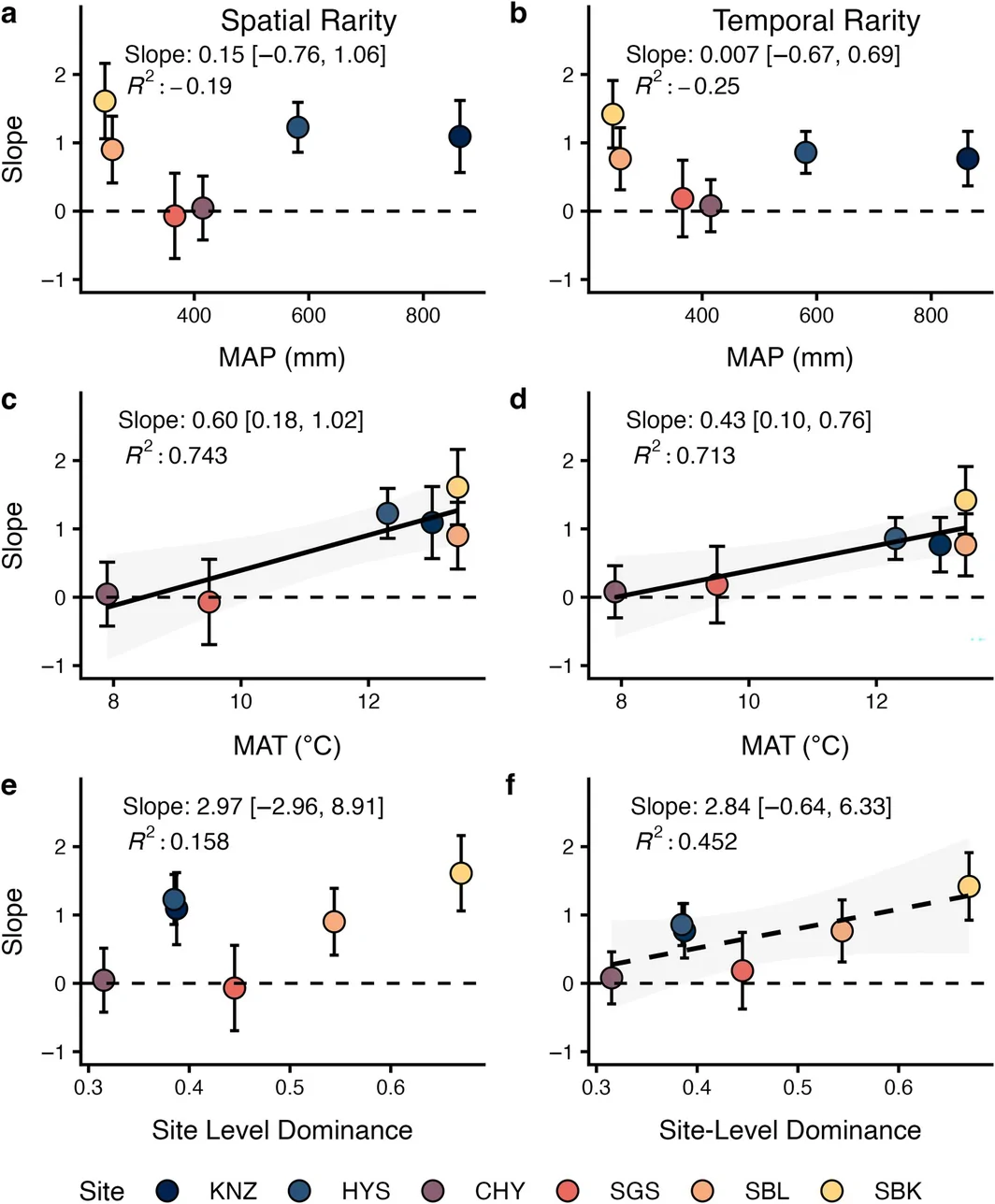
Slopes of linear models testing the effect of rarity on the drought response ratio as a function of mean annual precipitation (MAP) in millimeters (a, b), mean annual temperature (MAT) in degrees Celsius (c, d), and the site‐level dominance (e, f) calculated as the mean Berger–Parker dominance index at a site. Slope and adjusted *R* ^2^ values at the top of panels are from the relationship between the site‐level predictor (MAP, MAT, or dominance) and the rarity‐drought response ratio slope, with the 95% CI of the slope in brackets. Line type denotes significance: Solid black lines = significant (*p* < 0.05) and dashed black lines = marginally nonsignificant (0.05 < *p* < 0.1). Error bars on points show the 95% CI of rarity‐drought response ratio slopes, while point color indicates site. Linear model results are in Appendix S1: Table S9. Sites are as follows: CHY, High Plains Grasslands Research Center; HYS, Hays Agricultural Research Center; KNZ, Konza Prairie; SBK, Sevilleta Black Grama; SBL, Sevilleta Blue Grama; SGS, Short Grass Steppe.

The effects of spatial and temporal rarity on species responses to drought varied by site (Figure 2; Appendix S1: Table S8) but did not align with the gradient of site MAP or site aridity as expected (MAP: $S_{R}$: *F*_1,4_ = 0.203, *p* = 0.676; $T_{R}$: *F*_1,4_ = 0.0008, *p* = 0.979; site aridity: $S_{R}$: *F*_1,4_ = 0.025, *p* = 0.882; $T_{R}$: *F*_1,4_ = 0.044, *p* = 0.844). Rather, rare species increased under drought at the two mesic and two arid sites (KNZ, HYS, SBL, and SBK), while there was no relationship between rarity and drought response at intermediate precipitation sites (CHY, SGS) (Figure 4a,b; Appendix S1: Table S9). MAT predicted the slope between rarity and drought response ($S_{R}$: *F*_1,4_ = 15.46, *p* = 0.017; $T_{R}$: *F*_1,4_ = 13.44, *p* = 0.021; Figure 4c,d), likely because the two nonresponsive sites were the coldest, while the four responsive sites had similar temperatures (Figure 1b). Soil water holding capacity did not predict slopes during drought, but was aligned with legacy drought responses ($S_{R}$: *F*_1,4_ = 14.97, *p* = 0.018; $T_{R}$: *F*_1,4_ = 9.19, *p* = 0.039), although this relationship was likely driven by a single site, SGS, with both low field capacity and a small slope value (Appendix S1: Figure S12). Finally, site‐level dominance was overall not a significant predictor of slope but had a marginally nonsignificant effect when predicting the slope of the relationship between temporal rarity and drought response ($S_{R}$: *F*_1,4_ = 1.936, *p* = 0.236; $T_{R}$: *F*_1,4_ = 5.121, *p* = 0.086; Figure 4e,f).

## DISCUSSION

Extreme events can have idiosyncratic effects on species. We tested whether immediate and legacy responses of individual species to an experimental 4‐year extreme drought could be explained by species' long‐term abundance and persistence in the community. We found that local spatial and temporal rarity can predict some of the variation in species‐specific responses to extreme drought across six grassland sites. Specifically, spatially and temporally rare species benefited from both the immediate and legacy effects of drought, while common species declined (Figure 2). Spatial rarity and temporal rarity accounted for 14% and 15% of the variance in their respective models, indicating that while rarity is a meaningful predictor, other factors also drive species responses. Notably, changes to species' abundances remained altered for 4 years after drought, indicating that these changes are persistent and give evidence of species reordering within droughted plots.

By altering species abundances, extreme events like our experimentally imposed drought can alter the outcomes of existing community assembly processes (Thibault & Brown, 2008). Community assembly is a dynamic process where large‐scale environmental drivers and local stochastic (dispersal) and deterministic processes (species interactions) alter community composition and structure (HilleRisLambers et al., 2012). Although the perturbation of a drought had direct negative effects on species, especially abundant ones (Gherardi & Sala, 2015), local, deterministic species interactions likely played an important role in both reducing common species and increasing rare species. The decline in common species cover during drought may have resulted from an increase in negative frequency dependence, which can become stronger in drought due to greater competition with conspecifics (Diez et al., 2021) Although rarer species are expected to experience stronger negative frequency dependence than common species (Comita et al., 2010; Yenni et al., 2012), our findings suggest that interspecific competition was more limiting to many rare species than intraspecific competition as they were sensitive to changes in the abundance of common species (Adler et al., 2012). Our findings are consistent with species responses to natural drought in California annual grasslands (Prugh et al., 2018) and an experimental drought in a grazed pasture in the Swiss Jura mountains (Mariotte et al., 2013), which also attribute the drought‐induced increase in low abundance species to competitive release. In contrast, rare species were more likely to become locally extinct after an extreme natural drought in a Minnesota grassland (Tilman & Haddi, 1992).

Categorizing species into abundance typologies allowed us to consider the combined effects of spatial and temporal rarity on individual species drought responses. Species responses were often correlated during and after drought, especially for persistent species, indicating that strong inertia of these species or legacy effects of drought maintained their altered abundances (Gutbrodt et al., 2011; Müller & Bahn, 2022; Wilfahrt et al., 2023). *Common*, persistent species had a relatively uniform correlation (Figure 3a), while *sparse* persistent species had an asymmetric correlation, where species that increased during drought maintained elevated abundances after drought, but some species that declined during drought then increased after drought (Figure 3b). In contrast, immediate and legacy responses of sparse, *intermittent* species had a lower correlation, reflecting a much wider range of responses (Figure 3d). As a group, sparse intermittent species were mainly forbs and had a more even mix of annual and perennial species (Appendix S1: Figure S1), likely leading to more diverse functional traits and a wider breadth of drought tolerance and recovery strategies (Griffin‐Nolan et al., 2019; Matos et al., 2021).

We expected that the relationship between rarity and drought response would depend on modal rainfall conditions at a site, where rare species at mesic and intermediate precipitation sites experience greater competitive release, while species at arid sites tend to respond negatively regardless of rarity due to strong abiotic limitations (Louthan et al., 2015). Instead, the responses of rare species were more associated with species growth form and the overlap between common species phenology and the experimental summer drought. For example, annual species can persist through drought as early maturation allows them to escape drought (Volaire, 2018) and across sites, annual species on average increased during drought regardless of their rarity (Appendix S1: Figure S5). At cool, intermediate precipitation sites, many common species were early‐season C_3_ annuals (Figure 1e) and did not decline with drought, despite having demonstrated negative responses to water deficits (Ehleringer et al., 1997; Griffin‐Nolan et al., 2023), indicating that they grew when precipitation was available in the early season rather than during the experimentally imposed summer drought (Griffin‐Nolan et al., 2019; Knapp et al., 2020). Rarer species at cool, intermediate precipitation sites therefore had more variable responses to drought rather than experiencing a large release from competition with the decline of common perennial grasses, as at mesic and arid sites. Given that large‐scale geographic precipitation and productivity data cannot predict species‐level drought responses, our work points to plant community composition as the next target to investigate for predicting how common and rare species within a community respond during and after drought.

Changes to species relative abundances during the multiyear drought lasted for 4 years after the drought, indicating persistent shifts in community composition. While it is well documented that plant productivity declines during drought (Aguirre et al., 2021; Griffin‐Nolan et al., 2018; Smith et al., 2024), we showed that individual plant species responses can differ from this overall declining trend. We found that sparse and intermittent species increased in cover during drought, which may have contributed to stabilizing productivity losses over the duration of the 4 year drought in this experiment (Yu et al., 2025). Sparse and intermittent species had a wider range of drought responses and abilities to adapt and recover from drought. On the other hand, most common, persistent species did not recover as readily post‐drought. This work highlights the important role of subordinate species in maintaining ecosystem function in response to natural disturbances, such as prolonged drought. Indeed, without considering the relative productivity contributions of not only common but also rare species during and after drought, the important role of rare species in maintaining ecosystem functioning would be missed. Our work highlights that rare species can buffer grasslands against environmental stressors that result from climate change (Mouillot et al., 2013), and reveals how subordinate species help to stabilize productivity and functional diversity of a community.

## AUTHOR CONTRIBUTIONS

Carmen R. E. Watkins, Beatriz A. Aguirre, Lukas P. Bell‐Dereske, Y. Anny Chung, Megan E. Wilcots, Joan C. Dudney, and Cristina Portales‐Reyes contributed to analyses, Carmen R. E. Watkins and Lauren M. Hallett contributed to writing, and Melinda D. Smith provided data. The manuscript was developed through conversations with the entire author list and all authors discussed results, gave feedback, and contributed to manuscript edits.

## CONFLICT OF INTEREST STATEMENT

The authors declare no conflicts of interest.

## Supporting information


Appendix S1.

## Data Availability

Data (Watkins, Aguirre, Chung, Bell‐Dereske, et al., 2026) are available in the Environmental Data Initiative (EDI) repository at https://doi.org/10.6073/pasta/bb4f6e5346323e843382822f22b92449. Data from the Sevilleta EDGE sites were derived from Baur et al. (2024) and are available in the EDI repository at https://doi.org/10.6073/pasta/8d28fa9c31095c4c15836cbd3467f0d1. Code (Watkins, Aguirre, Chung, Wilcots, et al., 2026) is available in Zenodo at https://doi.org/10.5281/zenodo.20608187.
