# Supplementary material for: Drought conditions reorder plant communities through expansion of spatially sparse and temporally intermittent species
Source: Ecology. 2026 Sep 8;107(9):e70478. doi: 10.1002/ecy.70478 (PMC13553126; doi:10.1002/ecy.70478)
Supplement: Supplementary file 1 [file ECY-107-e70478-s001.pdf]

Appendix S1

Drought conditions reorder plant communities through expansion of spatially sparse and temporally intermittent species

Carmen R. E. Watkins, Beatriz A. Aguirre, Y. Anny Chung, Lukas P. Bell-Dereske, Lauren M. Hallett, David L. Hoover, Laureano A. Gherardi, Joan C. Dudney, Megan E. Wilcots, Jennifer A. Rudgers, Scott L. Collins, Melinda D. Smith, Forest Isbell, Tadashi Fukami, Hanan Farah, Cristina Portales-Reyes

Ecology

Supplementary Figures

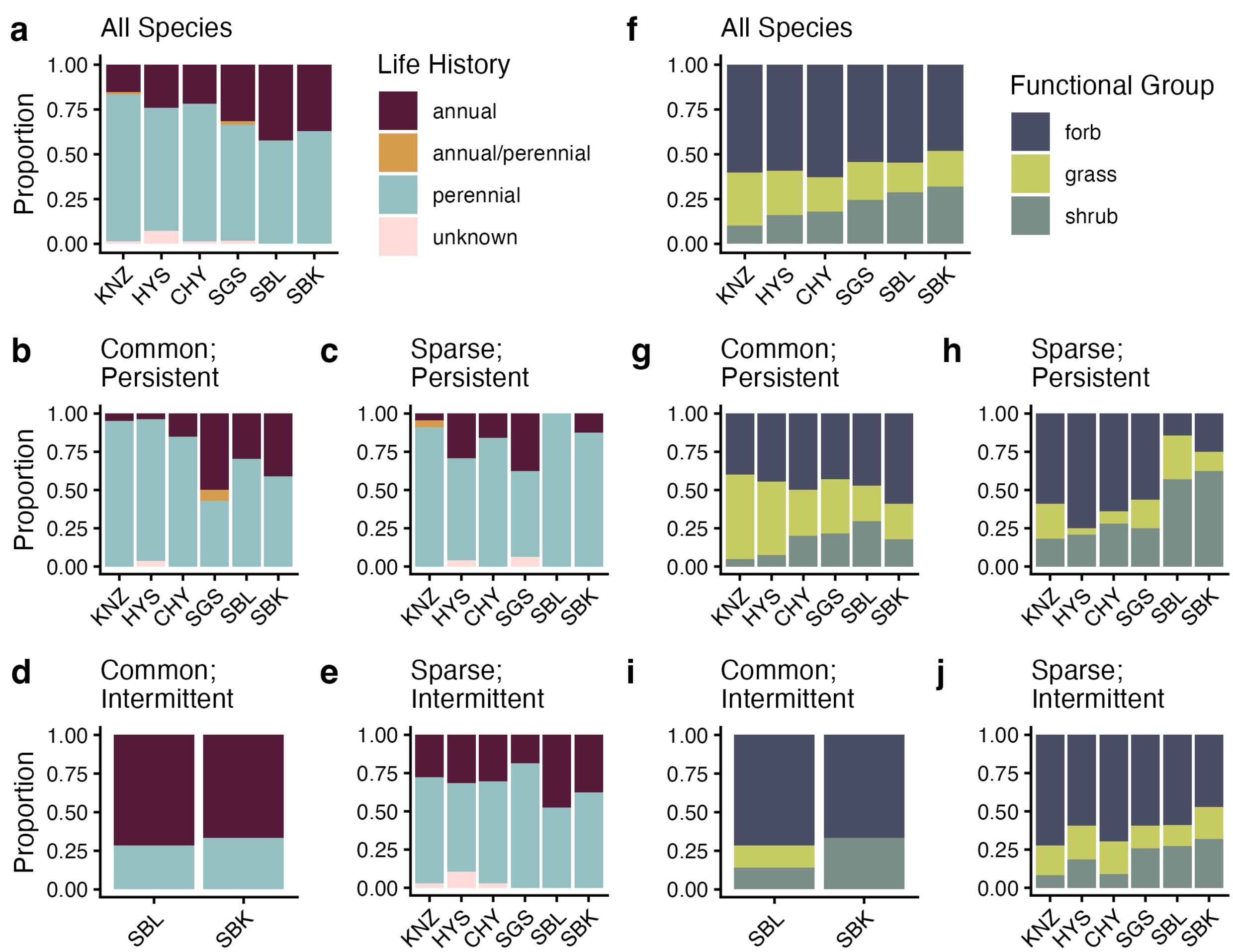

Figure S1. The proportion of species in life history or functional group categories for all species (a, f) and within each abundance typology: common, persistent species (b, g), sparse, persistent species (c, h), common, intermittent species (d, i), and sparse, intermittent species (e, j). Abundance typologies are defined by the combination of a species' spatial and temporal rarity,

where spatial rarity is categorized into common ($S_R < 0.25$) vs sparse ($S_R \geq 0.25$) species and temporal rarity into persistent ($T_R < 0.5$) vs intermittent ($T_R \geq 0.5$) species. Figure panel order aligns with Figure 3 in the main text to facilitate comparison. Sites are as follows, Konza Prairie (KNZ), Hays Agricultural Research Center (HYS), High Plains Grasslands Research Center (CHY), Short Grass Steppe (SGS), Sevilleta Blue Grama (SBL) and Sevilleta Black Grama (SBK).

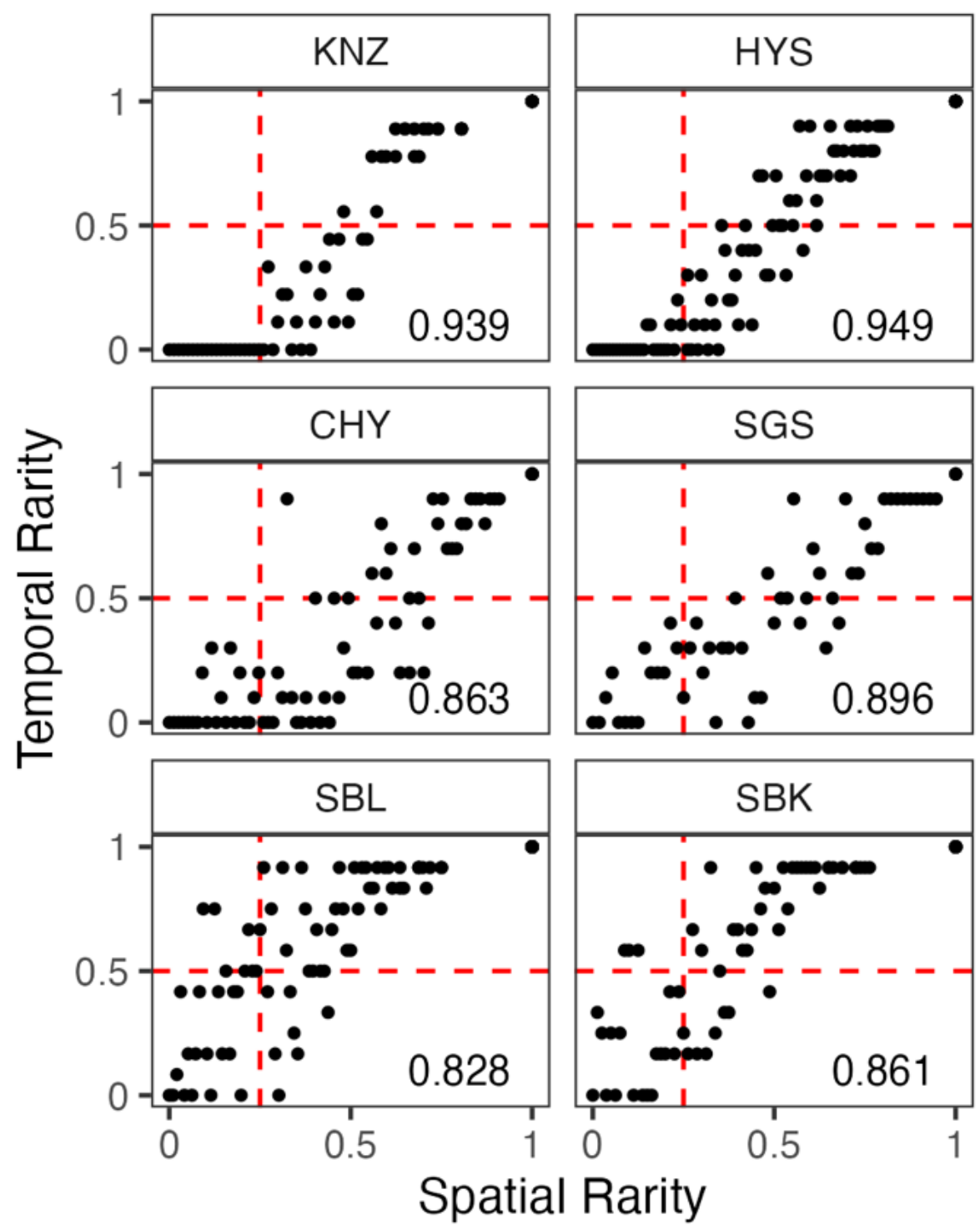

Figure S2. Points show the spatial and temporal rarity values for each species at each of the six sites in the experiment. Spatial and temporal rarity values were calculated from control plots at each site using Equations 1 and 2. Red dashed lines show the cutoffs for rarity categories: Spatially common species $S_R < 0.25$ and spatially sparse, $S_R \geq 0.25$. Temporally persistent species, $T_R < 0.5$ and temporally intermittent species $T_R \geq 0.5$. The Pearson's correlation coefficient between spatial and temporal rarity is in the bottom right corner of each panel. Sites are as follows, Konza Prairie (KNZ), Hays Agricultural Research Center (HYS), High Plains Grasslands Research Center (CHY), Short Grass Steppe (SGS), Sevilleta Blue Grama (SBL) and Sevilleta Black Grama (SBK).

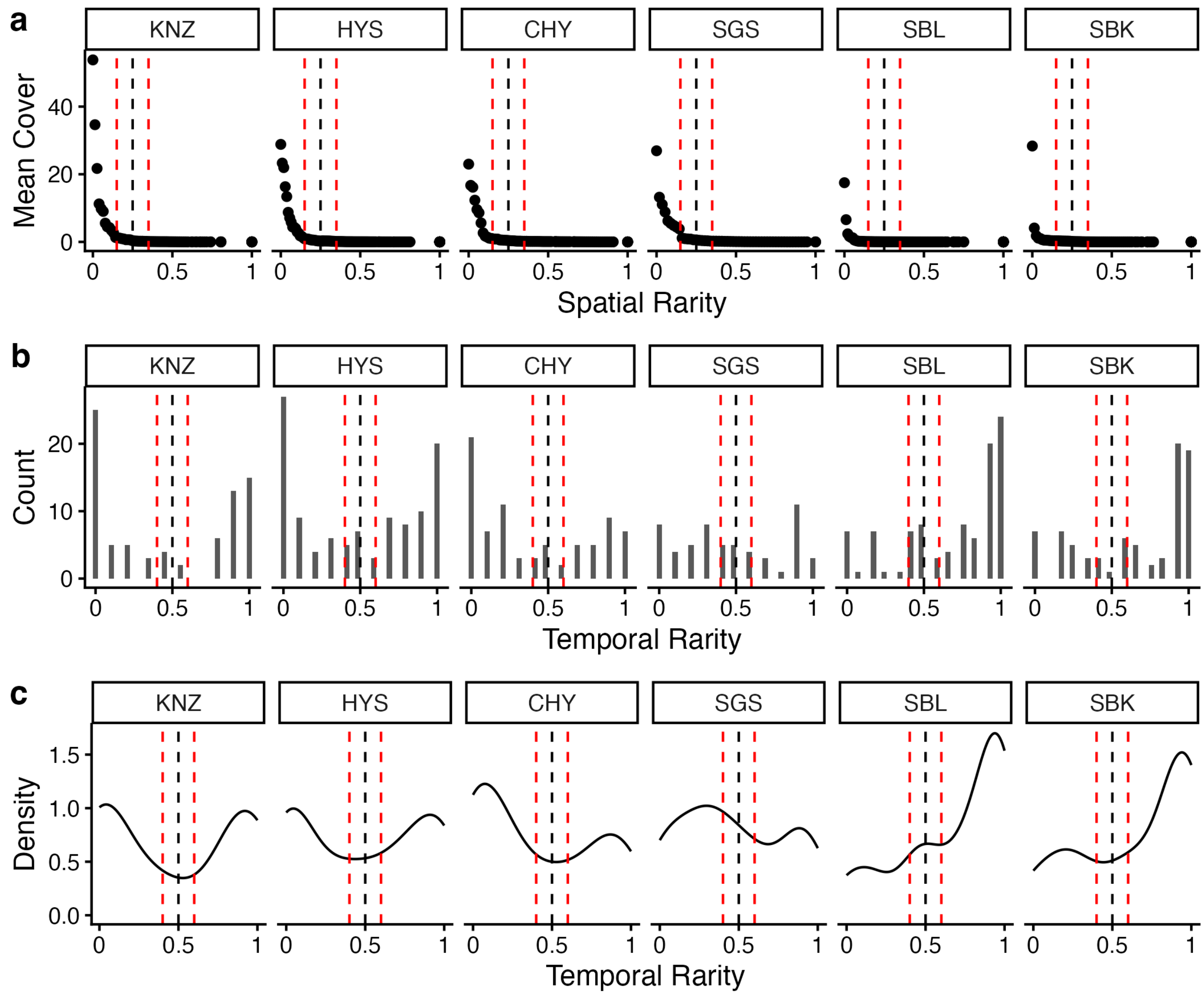

Figure S3. Rank abundance curves plotted using a species' mean cover in control plots and spatial rarity (a). The distribution and density plots of temporal rarity (b, c respectively) at each of the six experimental sites. Black dashed lines show the cutoff between spatially common species ($S_R < 0.25$) and sparse species ($S_R \geq 0.25$) in panel (a) and temporally persistent ($T_R < 0.5$) and intermittent ($T_R \geq 0.5$) species in panels (b) and (c). Red dashed lines show ± 0.10 of the rarity cutoffs that were used in sensitivity analyses. Sites are as follows, Konza Prairie (KNZ), Hays Agricultural Research Center (HYS), High Plains Grasslands Research Center (CHY), Short Grass Steppe (SGS), Sevilleta Blue Grama (SBL) and Sevilleta Black Grama (SBK).

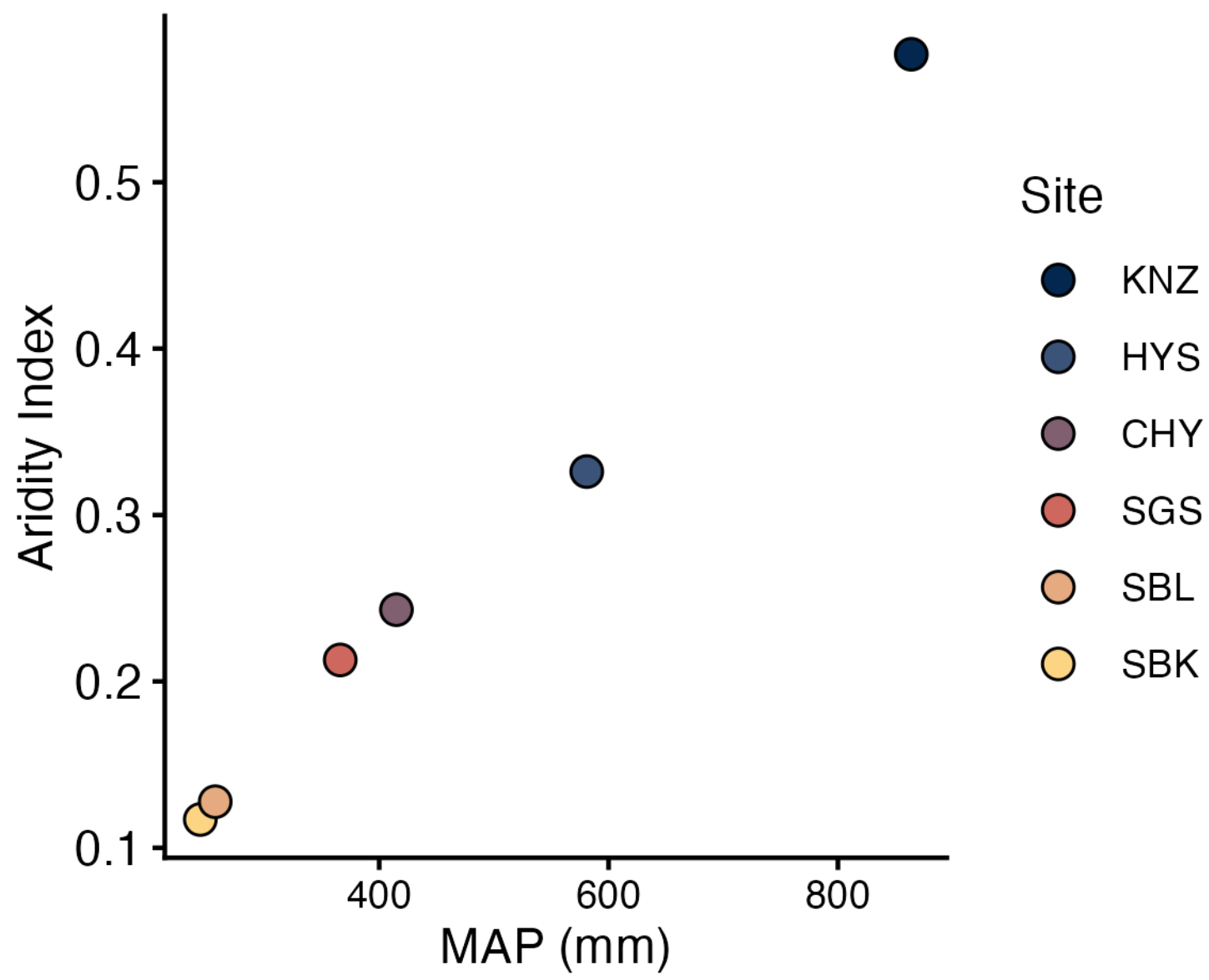

Figure S4. The aridity index of each site by the site mean annual precipitation (MAP) in mm. Points are colored by site. Sites are as follows, Konza Prairie (KNZ), Hays Agricultural Research Center (HYS), High Plains Grasslands Research Center (CHY), Short Grass Steppe (SGS), Sevilleta Blue Grama (SBL) and Sevilleta Black Grama (SBK).

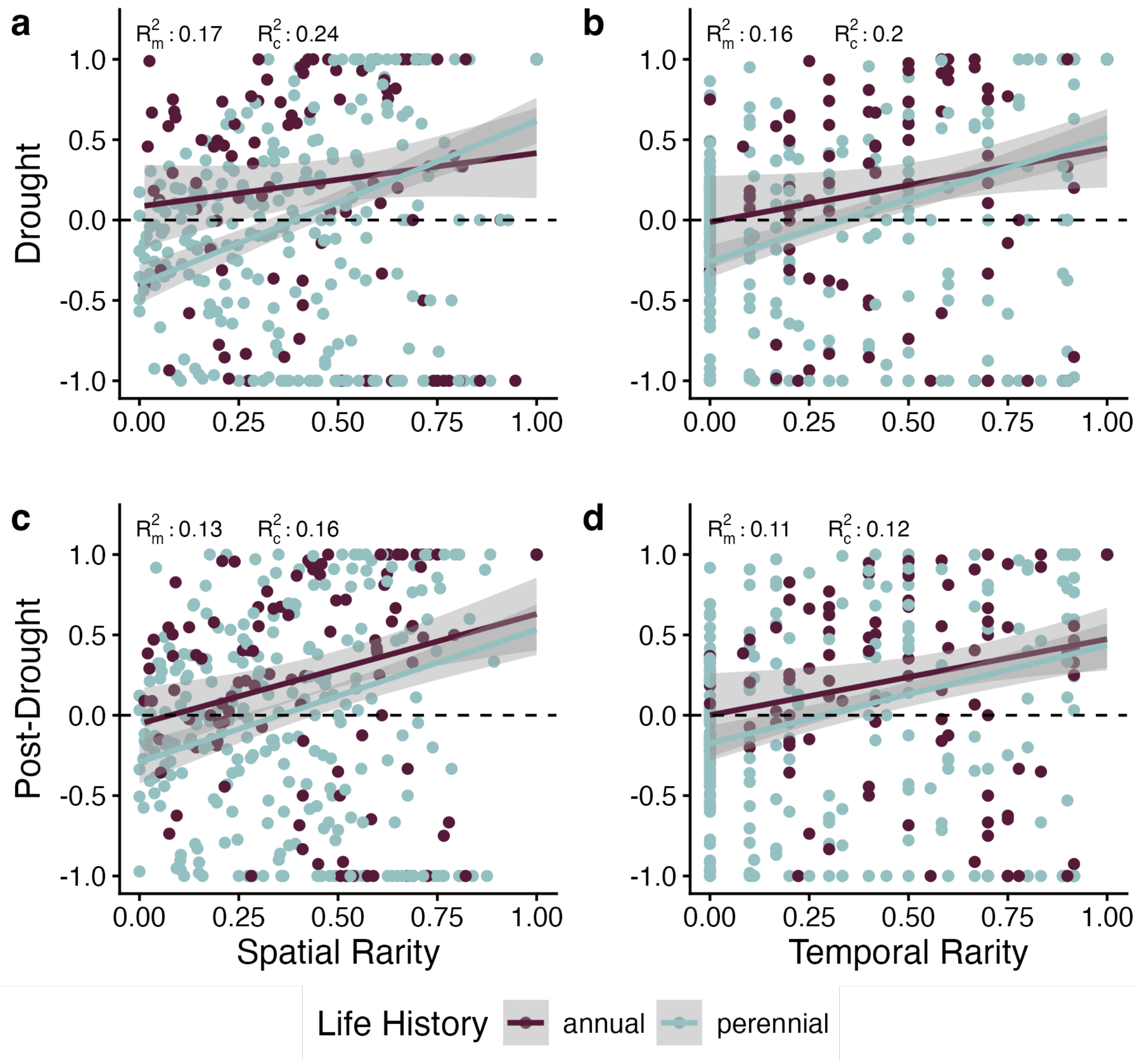

Figure S5. The effect of spatial (a, c) and temporal (b, d) rarity on a species' response ratio values during drought (a, b) and post-drought (c, d) for annual (purple) and perennial (blue) species. Each point shows one population of a species (i.e. species x site combination) and trend lines show the response across sites. Positive values of the response ratio indicate cover of an individual species is greater in drought plots than controls and negative values occur when cover is lower in drought plots than controls. Spatial or temporal rarity values near 1 indicate rare species and values near 0 indicate common species. The marginal R^2m is the variation explained by fixed effects alone, and the conditional R^2c is the variation explained by both fixed and random effects. Mixed effects model results are in Table S2 and estimated marginal slopes are in Table S3.

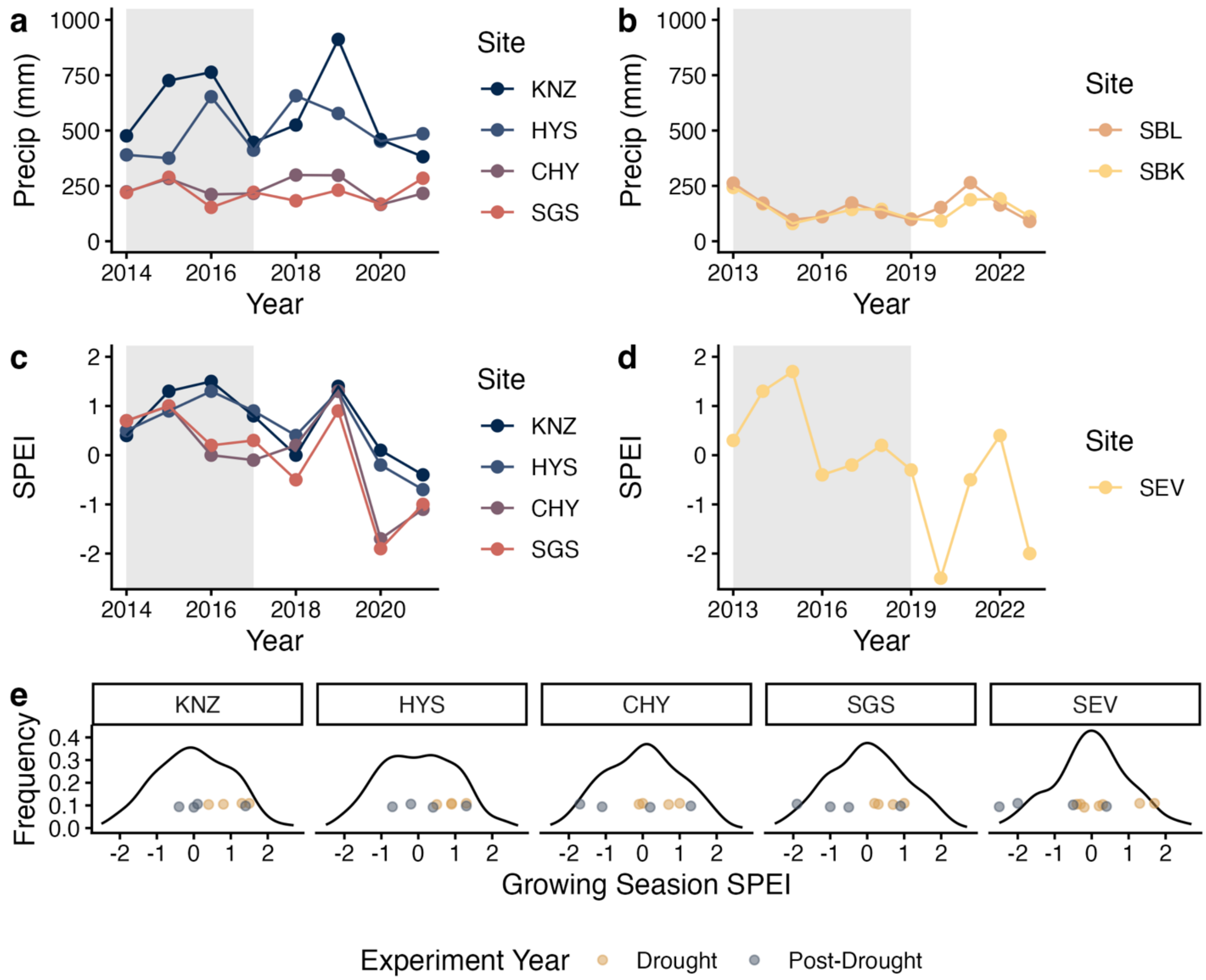

Figure S6. Growing season precipitation (a, b) and the growing season (6-month) standardized precipitation-evapotranspiration index (SPEI) (c, d) over the course of the experiment. The gray background indicates experimentally applied drought years: 2014 – 2017 for KNZ, HYS, CHY, and SGS and 2013 – 2019 for SBL and SBK. Panel d shows one line for both sites SBL and SBK, as they are located close together at the Sevilleta Long Term Ecological Research station (SEV) and thus have the same SPEI values. Points in panel (e) show SPEI values of experimental years plotted against the distribution of historical SPEI values from 1901 – 2024. (e). Here, experimental drought years are shown in orange and experimental post-drought years in purple. Points are jittered vertically by 0.01 to improve visibility of overlapping points, without changing interpretation of SPEI values. Sites are as follows, Konza Prairie (KNZ), Hays Agricultural Research Center (HYS), High Plains Grasslands Research Center (CHY), Short Grass Steppe (SGS), Sevilleta Blue Grama (SBL) and Sevilleta Black Grama (SBK).

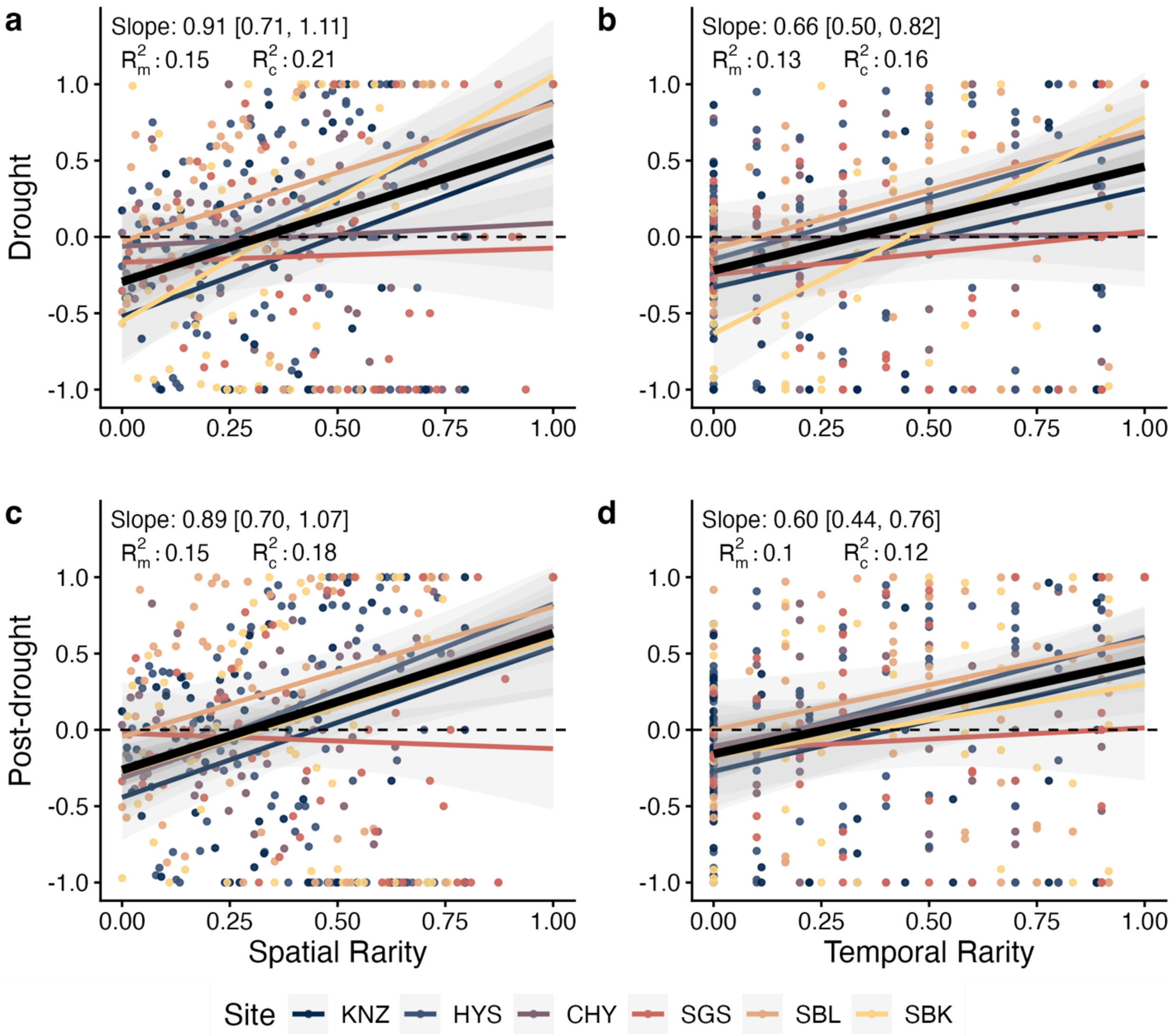

Figure S7. The relationship between spatial (a, c) or temporal (b, d) rarity and species response ratio values during drought (a, b) and post-drought (c, d) calculated *with unknown observations included in the dataset*. Compare to Figure 2 in the main text, where response ratios were calculated with unknown observations removed. Each point shows one population of a species (i.e. species x site combination). Black lines indicate the overall response across sites, while colored lines indicate individual site responses. Positive values of the response ratio indicate that the cover of an individual species is greater in drought plots than controls and negative values occur when cover is lower in drought plots than controls. Spatial or temporal rarity values near 1 indicate rare species and values near 0 indicate common species. Slope and R^2 values are from linear mixed effects models testing the effect of rarity on drought response across sites (Equation 4). Slope values are shown with the 95% CI in brackets. Marginal R^2_m = variation explained by fixed effects alone, Conditional R^2_c = variation explained by both fixed and random effects. Mixed effects model results are in Table S4. Sites are as follows, Konza Prairie (KNZ), Hays Agricultural Research Center (HYS), High Plains Grasslands Research Center

(CHY), Short Grass Steppe (SGS), Sevilleta Blue Grama (SBL) and Sevilleta Black Grama (SBK).

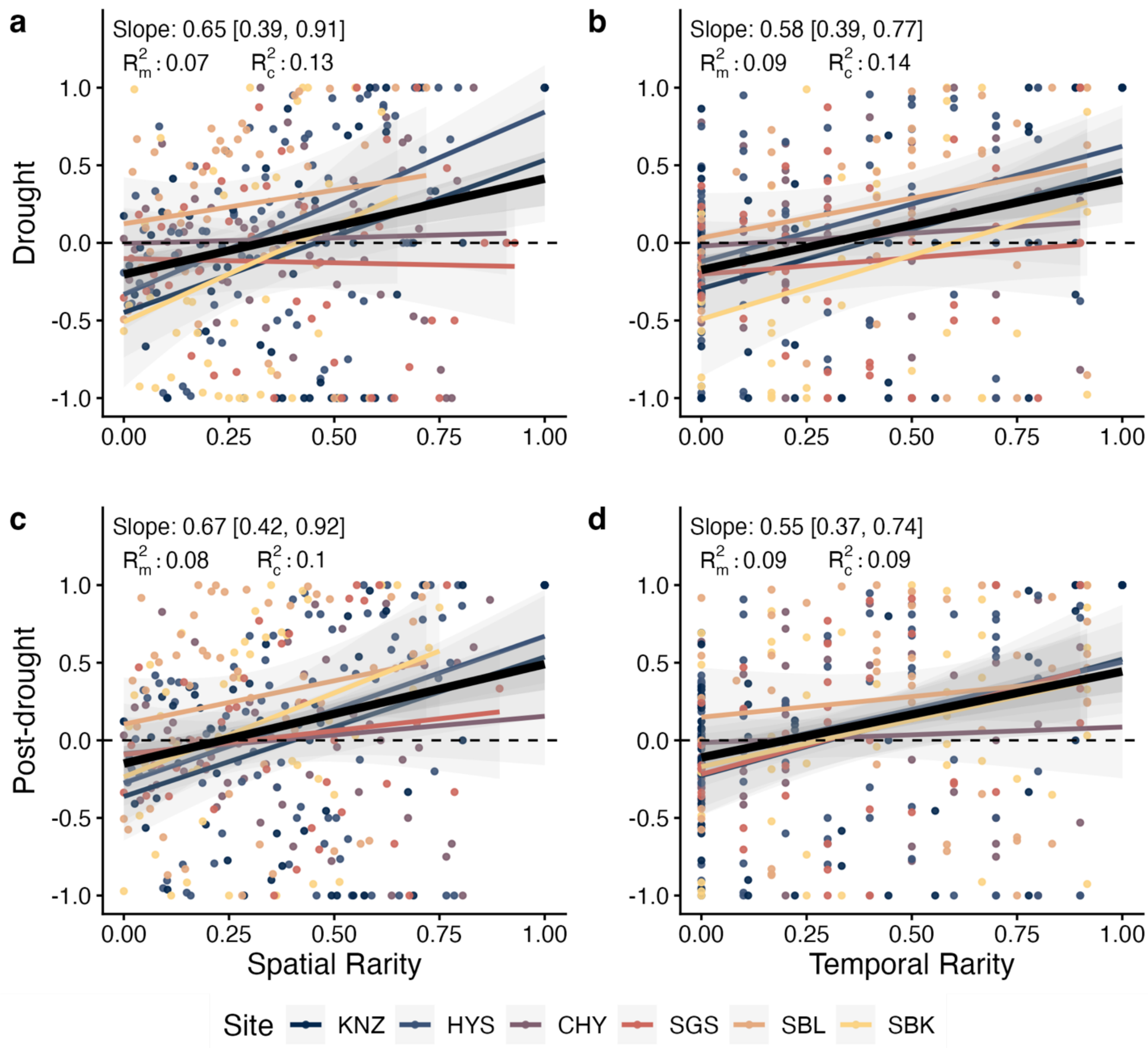

Figure S8. The relationship between spatial (a, c) or temporal (b, d) rarity and species response ratio values during drought (a, b) and post-drought (c, d) calculated *with transient species removed*. Transient species are defined as defined as not present in the 1-year pre-treatment data and gained or lost during the course of the experiment. Compare to Figure 2 in the main text, where response ratios were calculated with transient species present. Each point shows one population of a species (i.e. species x site combination). Black lines indicate the overall response across sites, while colored lines indicate individual site responses. Positive values of the response ratio indicate that the cover of an individual species is greater in drought plots than controls and negative values occur when cover is lower in drought plots than controls. Spatial or temporal rarity values near 1 indicate rare species and values near 0 indicate common species. Slope and

R^2 values are from linear mixed effects models testing the effect of rarity on drought response across sites (Equation 4). Slope values are shown with the 95% CI in brackets. Marginal R^2_m = variation explained by fixed effects alone, Conditional R^2_c = variation explained by both fixed and random effects. Mixed effects model results are in Table S5. Sites are as follows, Konza Prairie (KNZ), Hays Agricultural Research Center (HYS), High Plains Grasslands Research Center (CHY), Short Grass Steppe (SGS), Sevilleta Blue Grama (SBL) and Sevilleta Black Grama (SBK).

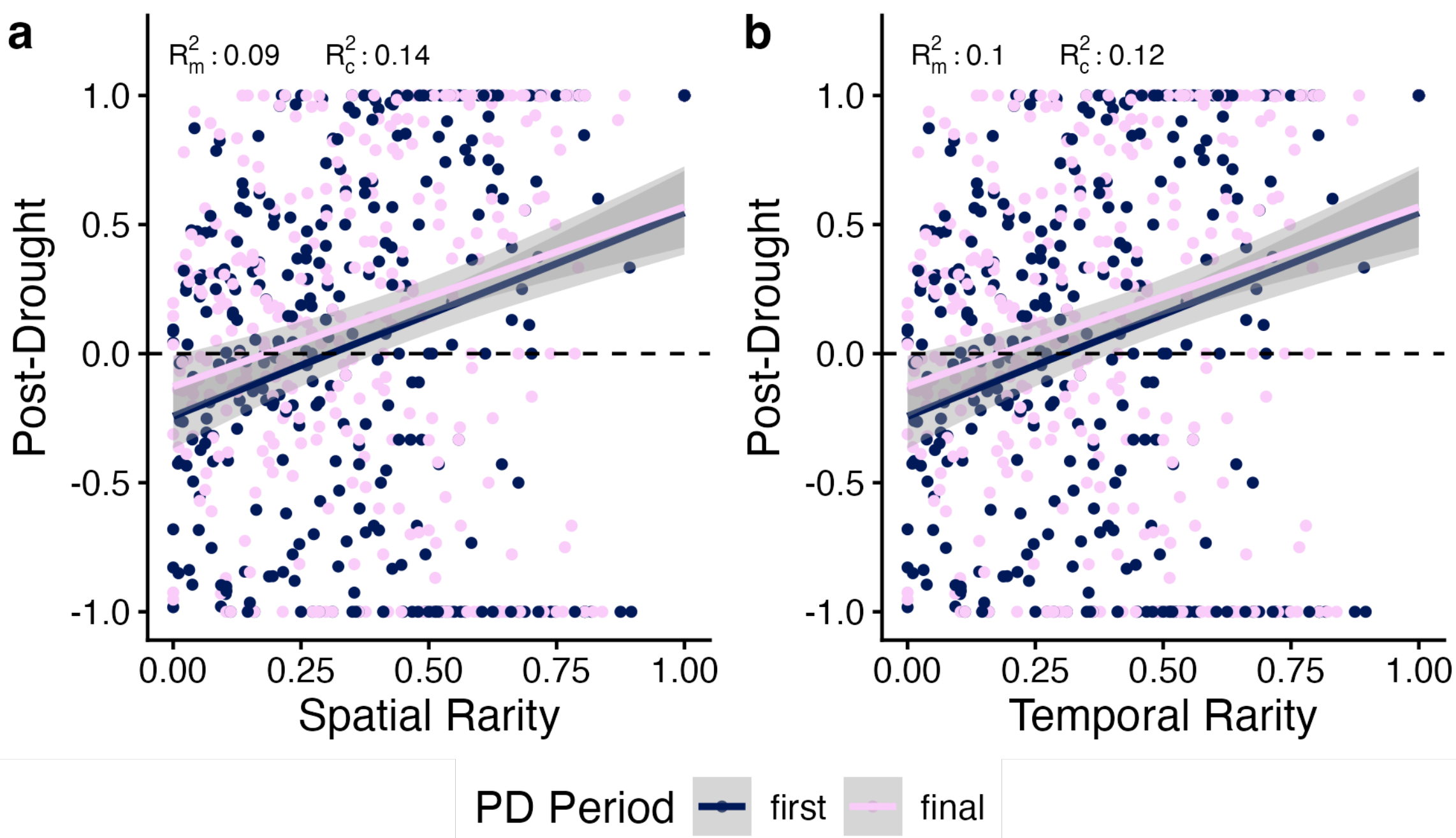

Figure S9. The effect of spatial rarity (a) and temporal rarity (b) on species response ratio values during the first and final two years of the four-year post-drought (PD) period. We split the post-drought period into halves to assess whether calculating a response ratio across the post-drought period is representative of recovery or if an early lack of recovery masks later trends. Line and point color indicates the first (navy) or final (pink) half of the post-drought period and each point shows one species population (species x site combination). Positive values of the response ratio indicate that the cover of an individual species is greater in drought plots than controls and negative values occur when cover is lower in drought plots than controls. Spatial or temporal rarity values near 1 indicate rare species and values near 0 indicate common species. R^2 values are from linear mixed effects models testing the effects of rarity and post-drought period on drought responses across sites. Marginal R^2_m = variation explained by fixed effects alone, Conditional R^2_c = variation explained by both fixed and random effects. Mixed effects model results are in Table S6.

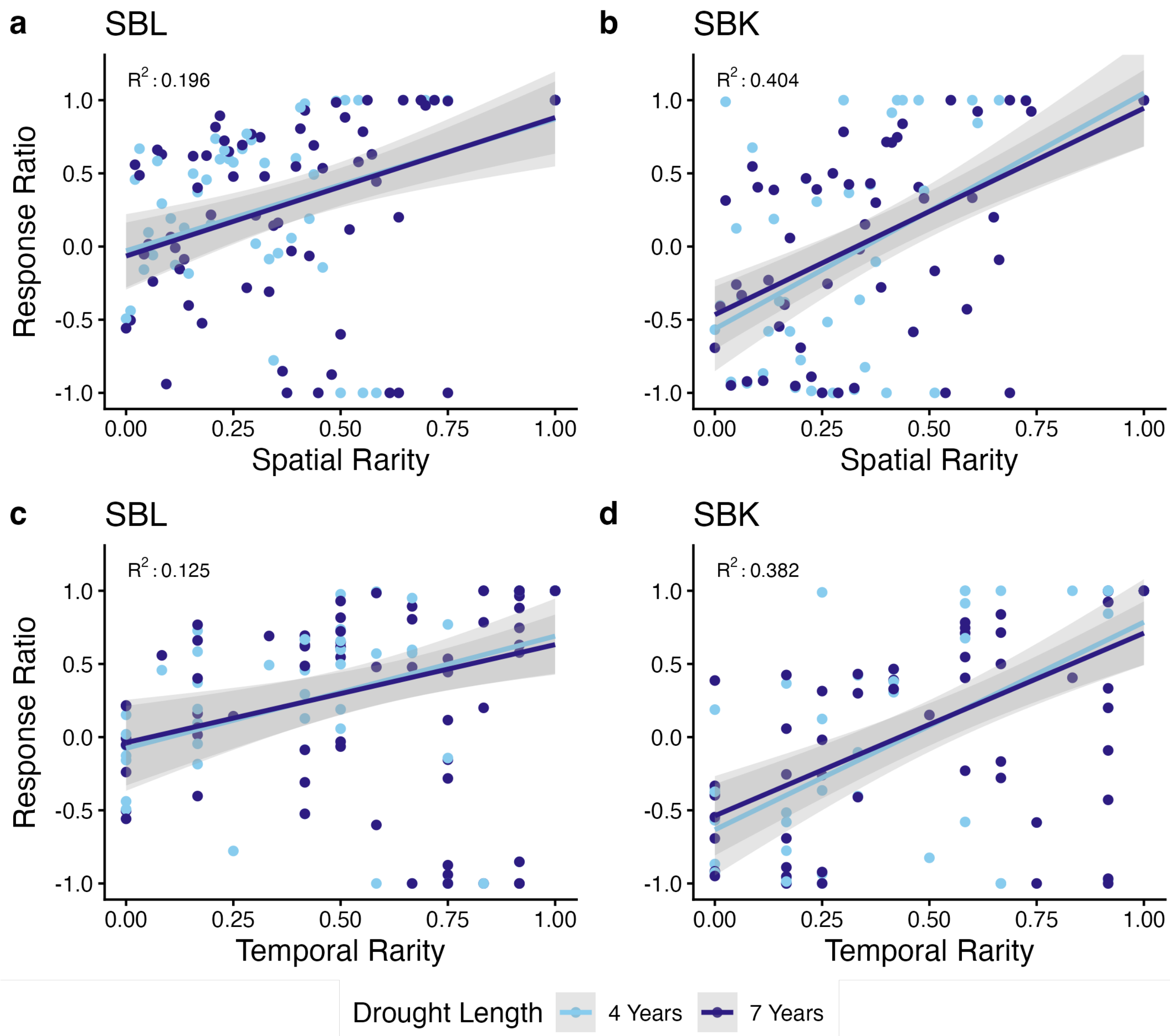

Figure S10. The effect of spatial (a, b) and temporal (c, d) rarity on a species response ratio values calculated over 4 years (light blue) or 7 years (dark blue) of drought at the Sevilleta Blue Grama (SBL) and Sevilleta Black Grama (SBK) sites. Each point shows one species population (species x site combination). Positive values of the response ratio indicate that the cover of an individual species is greater in drought plots than controls and negative values occur when cover is lower in drought plots than controls. Spatial or temporal rarity values near 1 indicate rare species and values near 0 indicate common species. R^2 values are from linear models testing the effects of rarity (continuous) and drought length (4 or 7 years) on drought responses separately at each site. Linear model results are in Table S7.

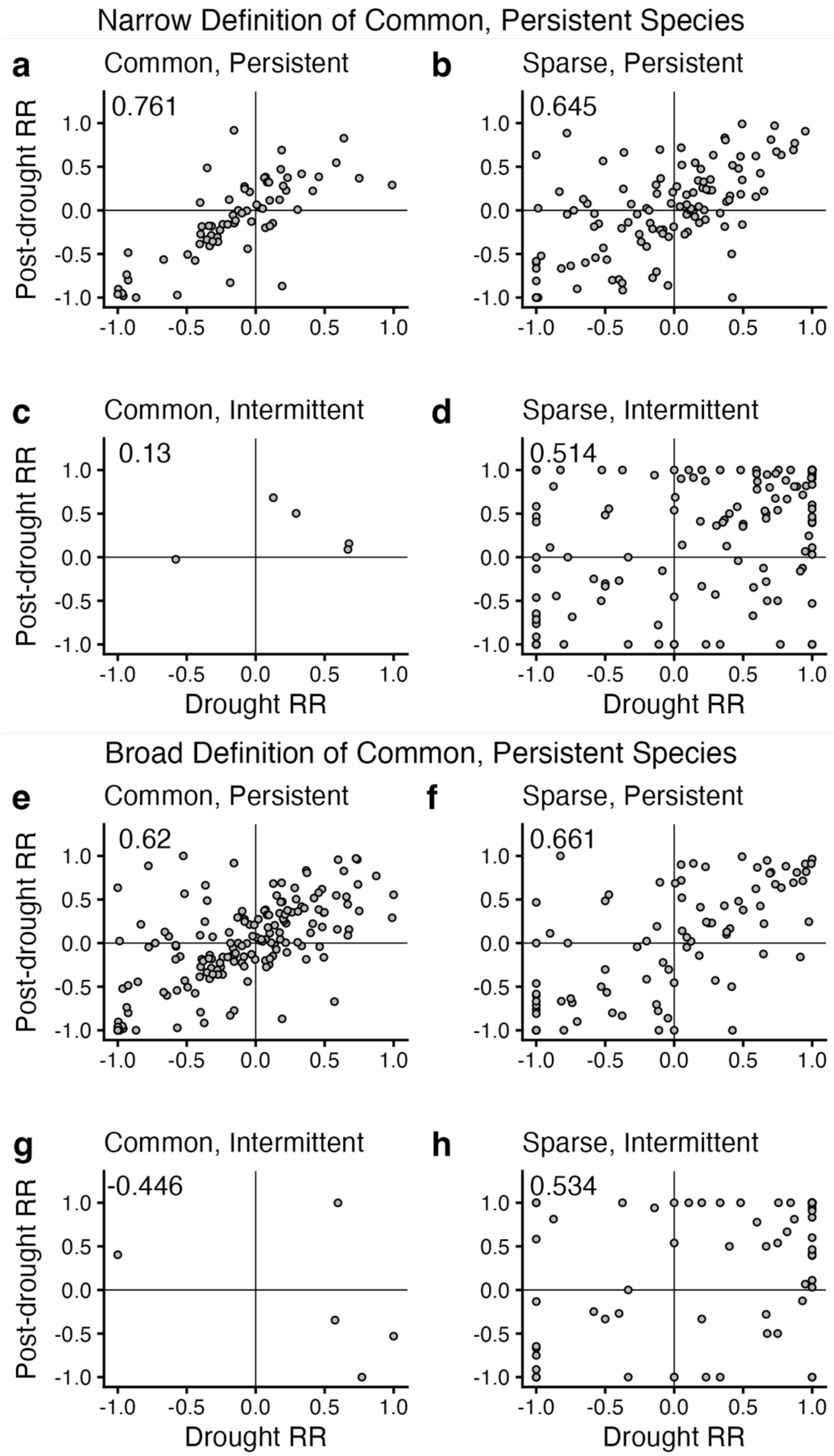

Figure S11. *Sensitivity analysis of abundance typology cutoffs*. The drought response ratio (RR) plotted against the post-drought response ratio for each species, separated into panels by the abundance typology of the species: common, persistent species (a, e), sparse, persistent species

(b, f), common, intermittent species (c, g), and sparse, intermittent species (d, h). Abundance typologies were defined by the combination of a species' spatial and temporal rarity, where spatial rarity was categorized into common ($S_R < 0.25$) vs sparse ($S_R \geq 0.25$) species and temporal rarity into persistent ($T_R < 0.5$) vs intermittent ($T_R \geq 0.5$) species in main text analyses. In the sensitivity analyses shown here, cutoffs for each category were adjusted by ± 0.10, such that common and persistent species were defined either more narrowly, by $S_R < 0.15$ and $T_R < 0.4$ (a-d), or more broadly, by $S_R < 0.35$, $T_R < 0.6$ (e-h). Each point shows one population of a species (i.e. species x site combination). Pearson's correlation coefficients for the relationship between drought and post-drought response ratios are shown in the top left corner of each panel. Compare to Figure 3 in the main text.

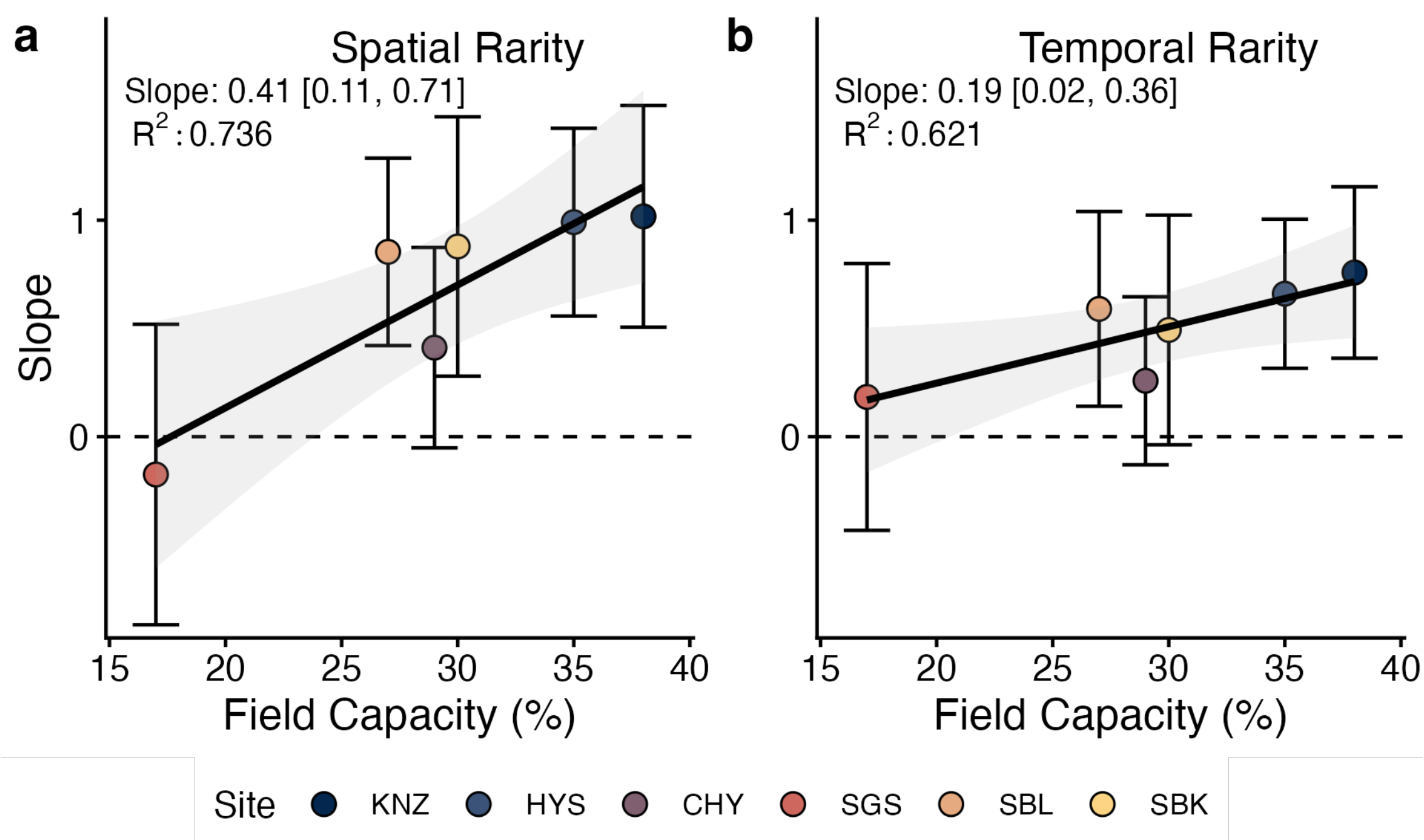

Figure S12. Slopes of linear models testing the effect of rarity on the *post-drought* response ratio as a function of soil field capacity for spatial (a) and temporal (b) rarity. Slope and adjusted R^2 values of the relationship between the site level predictor (field capacity) and the rarity – post-drought response ratio slope are shown at the top of panels with the 95% confidence interval of the slope in brackets. Line type denotes significance: solid black lines indicate a significant predictor ($p<0.05$). Error bars on points show the 95% confidence interval of rarity – post-drought response ratio slopes, while point color indicates site. Linear model results are in Table S9. Sites are as follows, Konza Prairie (KNZ), Hays Agricultural Research Center (HYS), High Plains Grasslands Research Center (CHY), Short Grass Steppe (SGS), Sevilleta Blue Grama (SBL) and Sevilleta Black Grama (SBK).

Supplementary Tables

Table S1. Site characteristics of the six grassland sites used in this study.

	KNZ	HYS	CHY	SGS	SBL	SBK
	Tallgrass prairie	Mixed-Grass prairie	Mixed-Grass prairie	Shortgrass prairie	Shortgrass prairie	Desert grassland
MAP (mm)[a]	864	581	415	366	257	244
MAT (C)[a]	13	12.3	7.9	9.5	13.4	13.4
Dom[b]	0.39	0.38	0.31	0.42	0.54	0.67
Soil FC (%)[c]	38	35	29	17	27	30
Example Species in each Abundance Typology						
Common, Persistent	*Andropogon gerardii, Sorghastrum nutans, Amorpha canescens, Ambrosia psilostachya*	*Bouteloua curtipendula, Schizachyrium scoparium, Sporobolus asper, Schrankia nutallii*	*Bouteloua gracilis, Carex Eleocharis, Pascopyrum smithii, Erigeron pumilis*	*Bouteloua gracilis, Elymus elymoides, Vulpia octoflora, Plantago patagonica*	*Bouteloua gracilis, Bouteloua eriopoda, Chamaesyce serpyllifolia, Salsola tragus*	*Bouteloua eriopoda, Sporobolus sp., Salsola tragus, Allionia incarnata,*
Sparse, Persistent	*Dalea purpurea, Asclepias viridis, Baptisia bracteata, Bouteloua gracilis*	*Sporobolus cryptandrus, Achillea millefolium, Lepidium densiflorum, Dalea purpurea*	*Artemesia dracunculus, Gutierrezia sarothrae, Potentilla pensylvanica, Bouteloua dactyloides*	*Aristida purpurea, Mirabilis linearis, Conyza canadensis, Ipomopsis laxiflora*	*Escobaria vivipara, Aristida purpurea, Dasyochloa pulchella, Zinnia grandiflora*	*Solanum elaeagnifolium, Oenothera caespitosa, Pleuraphis jamesii, Linum puberulum*
Sparse, Intermittent	*Poa pratensis, Lespedeza violacea, Carex gravida, Oxalis violacea*	*Koeleria macrantha, Hordeum pusilum, Draba reptans, Solidago canadensis*	*Poa pratensis, Bouteloua hirsuta, Draba nemorosa, Sisymbrium altissimum*	*Oxytropis sericea, Bouteloua hirsuta, Taraxacum officinale, Lactuca serriola*	*Tragus racemosus, Malacothrix fendleri, Bahia dissecta, Chenopodium incanum*	*Ipomopsis pumila, Amaranthus blitoides, Aristida purpurea, Opuntia phaeacantha*
Common, Intermittent	*NA*	*NA*	*NA*	*NA*	*Gilia mexicana, Eriastrum diffusum, Allionia incarnata*	*Chamaesyce serpyllifolia, Opuntia macrocenta, Phacelia crenulata*

Abbreviations: Sites are as follows, Konza Prairie (KNZ), Hays Agricultural Research Center (HYS), High Plains Grasslands Research Center (CHY), Short Grass Steppe (SGS), Sevilleta Blue Grama (SBL) and Sevilleta Black Grama (SBK). MAP = Mean annual precipitation, MAT = mean annual temperature, Dom = Berger parker dominance, FC = Soil field capacity.
[a] MAP and MAT were obtained from Griffin-Nolan et al. (2018). [b] Berger parker dominance was calculated in control plots. [c] Soil field capacity was estimated using hourly soil moisture data from 2012-2013 for SBL and SBK and from 2013-2015 in SGS, CHY, HYS, KNZ, obtained from Wilcox et al. (2023).

Table S2. Model coefficient table from separate linear mixed effects models for each period (drought or post-drought) and each type of rarity (spatial or temporal) with rarity (continuous) and life history (annual vs. perennial) as fixed effects predictors of the response ratio with a random effect of site (Equation 5).

Period	Rarity	Coefficient	Estimate	Std. Error	df	t value	Pr(>|t|)
Drought	Spatial	Intercept	0.09	0.14	36.57	0.64	0.526
		Rarity	0.27	0.20	386.38	1.33	0.184
		Perennial Life History	-0.52	0.13	387.73	-3.93	9.96×10^{-5}
		Rarity: Perennial Life History	0.79	0.24	386.54	3.37	8.43×10^{-4}
	Temporal	Intercept	0.02	0.14	85.34	0.16	0.877
		Rarity	0.36	0.2	386.8	1.79	0.074
		Perennial Life History	-0.29	0.14	387.62	-2.13	0.034
		Rarity: Perennial Life History	0.42	0.22	386.47	1.86	0.064
Post-Drought	Spatial	Intercept	-0.06	0.12	84.43	-0.47	0.640
		Rarity	0.64	0.19	407.61	3.27	0.001
		Perennial Life History	-0.25	0.13	407.10	-1.87	0.062
		Rarity: Perennial Life History	0.19	0.23	407.53	0.82	0.411
	Temporal	Intercept	0.02	0.14	169.49	0.14	0.891
		Rarity	0.43	0.2	395.47	2.16	0.031
		Perennial Life History	-0.2	0.14	406.65	-1.41	0.160
		Rarity: Perennial Life History	0.18	0.22	407.6	0.81	0.420

Abbreviations: Std. Error = standard error, df = degrees of freedom

Table S3. Estimated marginal slopes of the relationship between rarity and the response ratio for annual and perennial species.

Experimental Period	Rarity	Coefficient	Slope	Std. Error	Df	95% CI	t ratio	p-value
Drought	Spatial	Annual	0.27	0.20	386	-1.30, 0.67	-3.36	0.0009
		Perennial	1.06	0.12	384	0.83, 1.30		
	Temporal	Annual	0.36	0.20	387	-0.04, 0.76	-1.85	0.065
		Perennial	0.78	0.10	387	0.59, 0.97		
Post-Drought	Spatial	Annual	0.64	0.20	408	0.25, 1.02	-0.82	0.413
		Perennial	0.83	0.12	404	0.58, 1.07		
	Temporal	Annual	0.43	0.20	396	0.03, 0.82	-0.80	0.422
		Perennial	0.60	0.10	405	0.40, 0.80		

Abbreviations: Std. Error = standard error, df = degrees of freedom. Notes: t ratio and p-value columns are from a post-hoc test, testing for the difference between annual and perennial slopes.

Table S4. Fixed effects coefficients from linear mixed effects models testing the effect of rarity on a species response ratio during and after drought, calculated *with unknown observations included in the dataset*, with a random effect of site to account for differences in sites (Equation 4).

Experimental Period	Rarity Type	Coefficient	Estimate	Std. Error	df	t value	Pr(>|t|)
Drought	Spatial	Intercept	-0.30	0.09	8.91	-3.57	0.006
		Rarity	0.91	0.10	411.37	8.93	$< 2\times10^{-16}$
	Temporal	Intercept	-0.22	0.07	9.58	-3.06	0.013
		Rarity	0.66	0.08	414.42	7.88	2.91×10^{-14}
Post-Drought	Spatial	Intercept	-0.27	0.07	12.07	-3.76	0.003
		Rarity	0.89	0.09	465.58	9.42	$< 2\times10^{-16}$
	Temporal	Intercept	-0.16	0.06	12.95	-2.51	0.026
		Rarity	0.60	0.08	469.14	7.42	5.48×10^{-13}

Note: Compare to Table 1 in the main text. Abbreviations: Std. Error = standard error, df = degrees of freedom

Table S5. Fixed effects coefficients from linear mixed effects models testing the effect of rarity on a species response ratio during and after drought, calculated *with transient species removed*, with a random effect of site to account for differences in sites (Equation 4).

Experimental Period	Rarity	Coefficient	Estimate	Std. Error	df	t value	Pr(>|t|)
Drought	Spatial	Intercept	-0.22	0.08	10.31	-2.70	0.022
		Rarity	0.65	0.13	333	4.97	1.10×10^{-6}
	Temporal	Intercept	-0.19	0.07	7.85	-2.65	0.030
		Rarity	0.58	0.10	330.50	5.88	9.85×10^{-9}
Post-Drought	Spatial	Intercept	-0.16	0.07	15.39	-2.33	0.034
		Rarity	0.68	0.13	344.98	5.38	1.37×10^{-7}
	Temporal	Intercept	-0.11	0.05	345	-2.40	0.017
		Rarity	0.55	0.09	345	5.90	8.85×10^{-9}

Notes: Transient species are defined as defined as not present in the 1-year pre-treatment data and gained or lost during the course of the experiment. Compare to Table 1 in the main text. Abbreviations: Std. Error = standard error, df = degrees of freedom.

Table S6. Fixed effects coefficients from linear mixed effects models testing the additive effects of rarity and the post-drought period (first 2 years or final 2 years) on a species response ratio after drought, with a random effect of site to account for differences in sites.

Rarity	Coefficient	Estimate	Std. Error	df	t value	Pr(>|t|)
Spatial	Intercept	-0.16	0.08	10.15	-2.05	0.067
	Rarity	0.73	0.09	704.21	8.50	$<2 \times 10^{-16}$
	Post Drought Period: First	-0.07	0.05	704.51	-1.47	0.141
Temporal	Intercept	-0.10	0.06	11.21	-1.54	0.151
	Rarity	0.60	0.07	682.26	8.38	2.99×10^{-16}
	Post Drought Period: First	-0.07	0.05	705.12	-1.40	0.163

Abbreviations: Std. Error = standard error, df = degrees of freedom

Table S7. Model coefficients from linear models testing the additive effects of rarity (continuous) and drought length (4 or 7 years) on drought response ratios separately at each site, SBL and SBK.

Site	Rarity	Coefficient	Estimate	Std. Error	t value	Pr(>|t|)
SBL	Spatial	Intercept	-0.04	0.10	-0.41	0.680
		Rarity	0.93	0.15	6.00	1.65×10^{-8}
		Drought Length: 7 Years	-0.02	0.10	-0.16	0.870
	Temporal	Intercept	-0.05	0.11	-0.41	0.680
		Rarity	0.71	0.15	4.67	7.11×10^{-6}
		Drought Length: 7 Years	-0.02	0.10	-0.17	0.865
SBK	Spatial	Intercept	-0.52	0.11	-4.84	4.08×10^{-6}
		Rarity	1.50	0.17	8.94	7.93×10^{-15}
		Drought Length: 7 Years	0.01	0.11	0.08	0.933
	Temporal	Intercept	-0.58	0.12	-5.07	1.55×10^{-6}
		Rarity	1.33	0.15	8.55	6.34×10^{-14}
		Drought Length: 7 Years	0.002	0.11	0.01	0.989

Abbreviations: SBL = Sevilleta Blue Grama, SBK = Sevilleta Black Grama, Std. Error = standard error, df = degrees of freedom.

Table S8. Model coefficients from site-level linear models exploring the effect of temporal rarity on species response ratio.

Rarity	Period	Site	F_{df}	R^2	Coeff	Estimate *[95% CI]*	Std. Error	t value	p value
Spatial	Drought	KNZ	$17.14_{1, 65}$	0.20	Intercept	-0.54 *[-0.82, -0.25]*	0.14	-3.7	4.41×10^{-4}
					Rarity	1.09 *[0.57, 1.62]*	0.26	4.14	1.02×10^{-4}
		HYS	$44.45_{1, 94}$	0.31	Intercept	-0.36 *[-0.56, -0.15]*	0.1	-3.45	8.31×10^{-4}
					Rarity	1.23 *[0.86, 1.59]*	0.18	6.67	1.80×10^{-9}
		CHY	$0.04_{1, 68}$	-0.01	Intercept	-0.01 *[-0.26, 0.24]*	0.13	-0.1	0.923
					Rarity	0.05 *[-0.42, 0.51]*	0.23	0.2	0.846
		SGS	$0.05_{1, 50}$	-0.02	Intercept	-0.12 *[-0.45, 0.22]*	0.17	-0.69	0.490
					Rarity	-0.07 *[-0.69, 0.56]*	0.31	-0.22	0.825
		SBL	$13.62_{1, 59}$	0.17	Intercept	-0.03 *[-0.28, 0.22]*	0.13	-0.23	0.816
					Rarity	0.9 *[0.41, 1.39]*	0.24	3.69	4.90×10^{-4}
		SBK	$34.38_{1, 51}$	0.39	Intercept	-0.56 *[-0.85, -0.27]*	0.14	-3.91	2.72×10^{-4}
					Rarity	1.61 *[1.06, 2.16]*	0.27	5.86	3.35×10^{-7}
	Post-Drought	KNZ	$15.77_{1, 64}$	0.19	Intercept	-0.45 *[-0.74, -0.16]*	0.14	-3.15	0.002
					Rarity	1.02 *[0.51, 1.53]*	0.26	3.97	1.84×10^{-4}
		HYS	$20.58_{1, 92}$	0.17	Intercept	-0.3 *[-0.53, -0.06]*	0.12	-2.53	0.013

					Rarity	0.99 *[0.56, 1.42]*	0.22	4.54	1.73×10^{-5}
		CHY	$3.14_{1, 69}$	0.03	Intercept	-0.15 *[-0.41, 0.1]*	0.13	-1.2	0.235
					Rarity	0.41 *[-0.05, 0.87]*	0.23	1.77	0.081
		SGS	$0.26_{1, 45}$	-0.02	Intercept	0.01 *[-0.34, 0.36]*	0.17	0.05	0.961
					Rarity	-0.18 *[-0.87, 0.52]*	0.34	-0.51	0.614
		SBL	$15.4_{1, 80}$	0.15	Intercept	-0.05 *[-0.3, 0.21]*	0.13	-0.35	0.727
					Rarity	0.85 *[0.42, 1.29]*	0.22	3.92	1.83×10^{-4}
		SBK	$8.59_{1, 63}$	0.11	Intercept	-0.28 *[-0.6, 0.04]*	0.16	-1.74	0.087
					Rarity	0.88 *[0.28, 1.48]*	0.3	2.93	0.005
Temporal	Drought	KNZ	$14.82_{1, 65}$	0.17	Intercept	-0.34 *[-0.56, -0.11]*	0.11	-3	0.004
					Rarity	0.77 *[0.37, 1.17]*	0.2	3.85	2.73×10^{-4}
		HYS	$30.95_{1, 94}$	0.24	Intercept	-0.15 *[-0.32, 0.03]*	0.09	-1.66	0.100
					Rarity	0.86 *[0.55, 1.17]*	0.15	5.56	2.48×10^{-7}
		CHY	$0.17_{1, 68}$	-0.01	Intercept	-0.02 *[-0.2, 0.17]*	0.09	-0.2	0.842
					Rarity	0.08 *[-0.3, 0.46]*	0.19	0.42	0.678
		SGS	$0.44_{1, 50}$	-0.01	Intercept	-0.23 *[-0.52, 0.07]*	0.15	-1.55	0.127
					Rarity	0.18 *[-0.38, 0.75]*	0.28	0.66	0.511
		SBL	$11.41_{1, 59}$	0.15	Intercept	-0.08 *[-0.37, 0.21]*	0.15	-0.53	0.600
					Rarity	0.77 *[0.31, 1.22]*	0.23	3.38	0.001
		SBK	$33.25_{1, 51}$	0.38	Intercept	-0.63 *[-0.95, -0.32]*	0.16	-4.07	1.66×10^{-4}
					Rarity	1.42 *[0.92, 1.91]*	0.25	5.77	4.75×10^{-7}
	Post-Drought	KNZ	$14.62_{1, 64}$	0.17	Intercept	-0.27 *[-0.5, -0.05]*	0.11	-2.46	0.017
					Rarity	0.76 *[0.36, 1.15]*	0.2	3.82	3.00×10^{-4}
		HYS	$14.48_{1, 92}$	0.13	Intercept	-0.12 *[-0.31, 0.07]*	0.1	-1.26	0.212
					Rarity	0.66 *[0.32, 1]*	0.17	3.81	2.55×10^{-4}
		CHY	$1.76_{1, 69}$	0.01	Intercept	-0.05 *[-0.24, 0.14]*	0.1	-0.53	0.599
					Rarity	0.26 *[-0.13, 0.65]*	0.19	1.33	0.189
		SGS	$0.36_{1, 45}$	-0.01	Intercept	-0.14 *[-0.44, 0.16]*	0.15	-0.92	0.364
					Rarity	0.18 *[-0.43, 0.8]*	0.31	0.6	0.552
		SBL	$6.82_{1, 80}$	0.07	Intercept	0 *[-0.33, 0.32]*	0.16	-0.01	0.995
					Rarity	0.59 *[0.14, 1.04]*	0.23	2.61	0.011
		SBK	$3.45_{1, 63}$	0.04	Intercept	-0.18 *[-0.53, 0.18]*	0.18	-0.98	0.333
					Rarity	0.49 *[-0.04, 1.02]*	0.27	1.86	0.068

Abbreviations: Coeff = coefficients, Std. Error = standard error, df = degrees of freedom. Sites are as follows, Konza Prairie (KNZ), Hays Agricultural Research Center (HYS), High Plains Grasslands Research Center (CHY), Short Grass Steppe (SGS), Sevilleta Blue Grama (SBL) and Sevilleta Black Grama (SBK).

Table S9. Model coefficients for separate linear models testing whether site mean annual precipitation (mm), site mean annual temperature (°C), a site-level dominance index, the aridity index, or soil field capacity (%) predict the slope of the relationship between rarity and the response ratio during and after drought.

Period	Rarity	Predictor	Coefficient	Estimate	Std. Error	t value	Pr(>|t|)
Drought	Spatial	Precipitation	Intercept	0.8	0.3	2.68	0.055
			Slope	0.15	0.33	0.45	0.676
		Temperature	Intercept	0.8	0.14	5.76	0.005
			Slope	0.6	0.15	3.93	0.017
		Dominance	Intercept	-0.56	1.01	-0.55	0.609
			Slope	2.97	2.14	1.39	0.236
		Aridity	Intercept	0.72	0.61	1.18	0.303
			Slope	0.31	1.97	0.16	0.882
		Soil Field Capacity	Intercept	0.8	0.23	3.49	0.025
			Slope	0.45	0.25	1.77	0.151
	Temporal	Precipitation	Intercept	0.68	0.22	3.04	0.038
			Slope	0.01	0.24	0.03	0.979
		Temperature	Intercept	0.68	0.11	6.35	0.003
			Slope	0.43	0.12	3.67	0.021
		Dominance	Intercept	-0.62	0.59	-1.05	0.355
			Slope	2.84	1.26	2.26	0.086
		Aridity	Intercept	0.76	0.44	1.72	0.161
			Slope	-0.3	1.43	-0.21	0.844
		Soil Field Capacity	Intercept	0.68	0.2	3.45	0.026
			Slope	0.23	0.22	1.07	0.346
Post-Drought	Spatial	Precipitation	Intercept	0.66	0.19	3.45	0.026
			Slope	0.2	0.21	0.94	0.402
		Temperature	Intercept	0.66	0.14	4.87	0.008
			Slope	0.36	0.15	2.39	0.075
		Dominance	Intercept	0.33	0.83	0.4	0.709
			Slope	0.72	1.77	0.41	0.705
		Aridity	Intercept	0.44	0.4	1.09	0.337
			Slope	0.84	1.3	0.65	0.553
		Soil Field Capacity	Intercept	0.66	0.1	6.8	0.002
			Slope	0.41	0.11	3.87	0.018
	Temporal	Precipitation	Intercept	0.49	0.08	6.03	0.004
			Slope	0.14	0.09	1.59	0.187
		Temperature	Intercept	0.49	0.06	8.36	0.001
			Slope	0.19	0.06	2.92	0.043
		Dominance	Intercept	0.41	0.41	0.99	0.380
			Slope	0.18	0.88	0.2	0.848
		Aridity	Intercept	0.3	0.18	1.73	0.159
			Slope	0.7	0.57	1.22	0.289
		Soil Field Capacity	Intercept	0.49	0.06	8.58	0.001
			Slope	0.19	0.06	3.03	0.039

Abbreviations: Std. Error = standard error.

References

Griffin-Nolan, Robert J., Charles J. W. Carroll, Elsie M. Denton, et al. 2018. "Legacy Effects of a Regional Drought on Aboveground Net Primary Production in Six Central US Grasslands." *Plant Ecology* 219 (5): 505–15. https://doi.org/10.1007/s11258-018-0813-7.

Wilcox, Kevin R., Scott L. Collins, Alan K. Knapp, et al. 2023. "Assessing Carbon Storage Capacity and Saturation across Six Central US Grasslands Using Data–Model Integration." *Biogeosciences* 20 (13): 2707–25. https://doi.org/10.5194/bg-20-2707-2023.
